# Supplementary material for: A photo-triggered self-accelerated nanoplatform for multifunctional image-guided combination cancer immunotherapy
Source: Nat Commun. 2023 Aug 25;14:5216. doi: 10.1038/s41467-023-40996-2 (PMC10457322; doi:10.1038/s41467-023-40996-2)
Supplement: Supplementary file 1 — Supplementary Information [file 41467_2023_40996_MOESM1_ESM.pdf]

Supplementary information for

A photo-triggered self-accelerated nanoplatform for multifunctional image-guided combination cancer immunotherapy

Xiaoying Kang,[1,4] Yuan Zhang,[1,4] Jianwen Song,[1] Lu Wang,[2] Wen Li,[2]* Ji Qi[1]* & Ben Zhong Tang[3]*

[1]State Key Laboratory of Medicinal Chemical Biology, Frontiers Science Center for Cell Responses, Key Laboratory of Bioactive Materials, Ministry of Education, and College of Life Sciences, Nankai University, Tianjin 300071, China.

[2]Tianjin Key Laboratory of Biomedical Materials and Key Laboratory of Biomaterials and Nanotechnology for Cancer Immunotherapy, Institute of Biomedical Engineering, Chinese Academy of Medical Sciences and Peking Union Medical College, Tianjin 300192, China.

[3]School of Science and Engineering, Shenzhen Institute of Aggregate Science and Technology, The Chinese University of Hong Kong, Shenzhen, Guangdong 518172, China.

[4]These authors contributed equally: Xiaoying Kang, Yuan Zhang.

*Corresponding authors. e-mail: liwen@bme.pumc.edu.cn (W.L.); qiji@nankai.edu.cn (J.Q.); tangbenz@cuhk.edu.cn (B.Z.T.)

Supplementary Methods

Synthetic processes

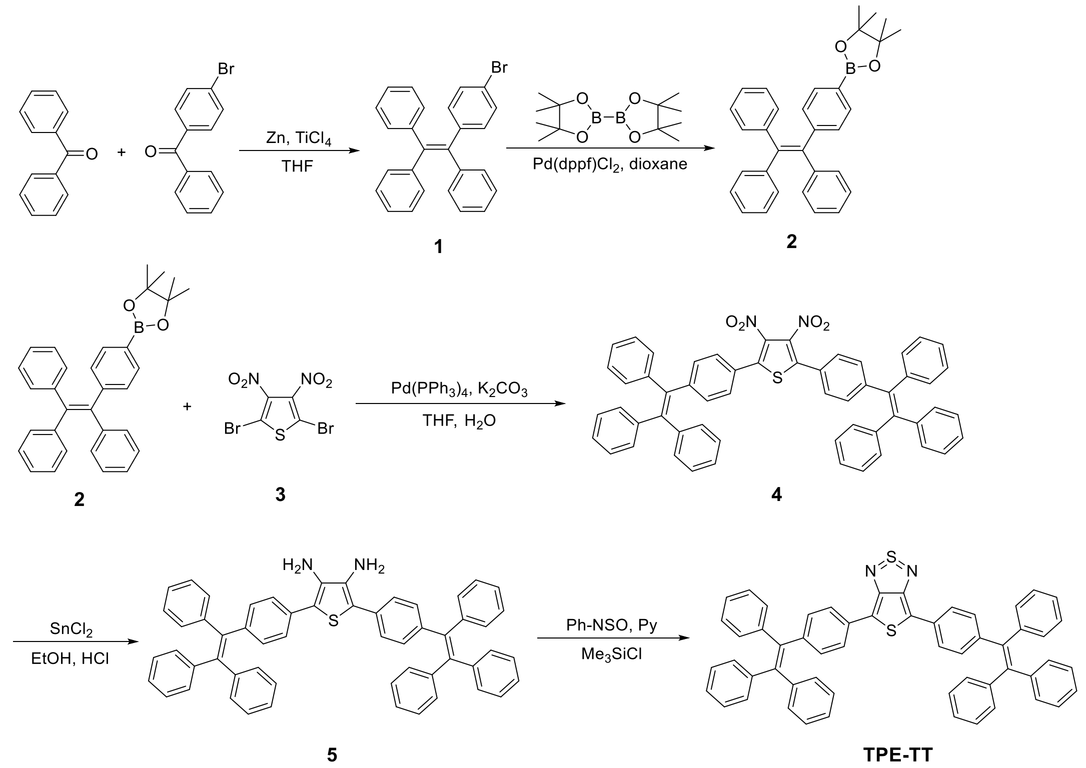

Supplementary Fig. 1. Synthetic route to TPE-TT.

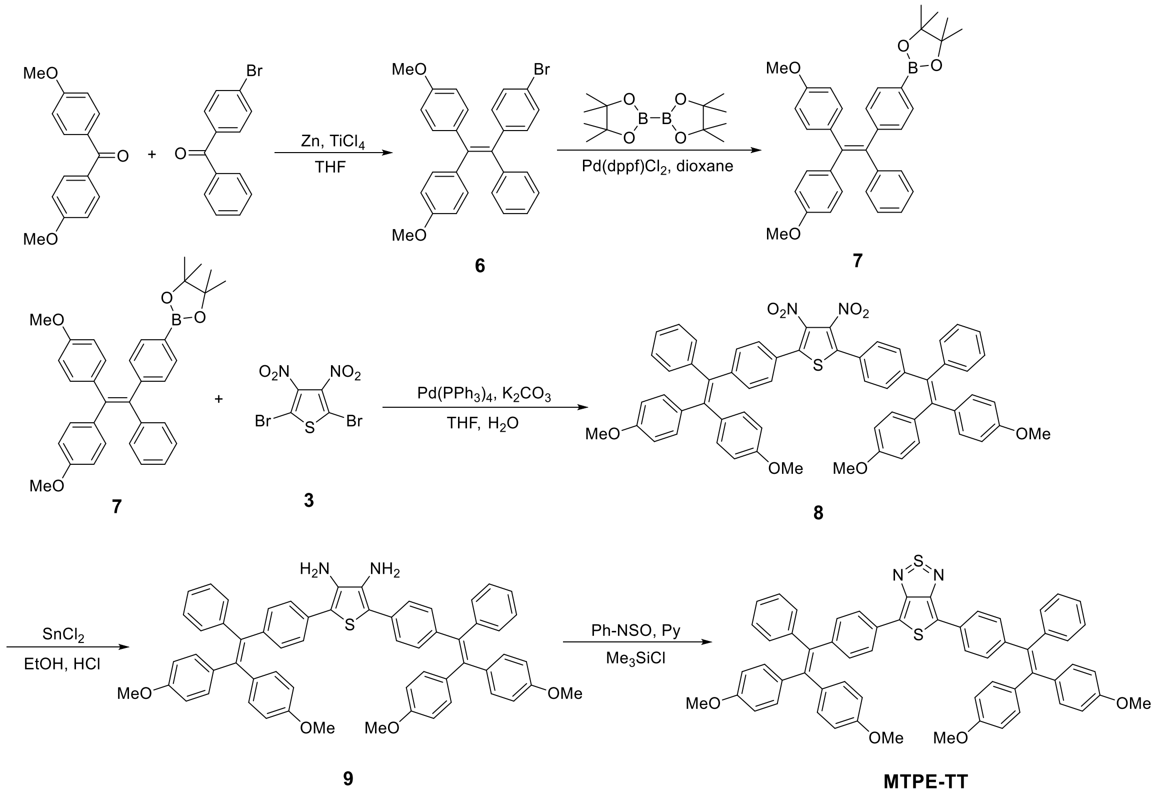

Supplementary Fig. 2. Synthetic route to MTPE-TT.

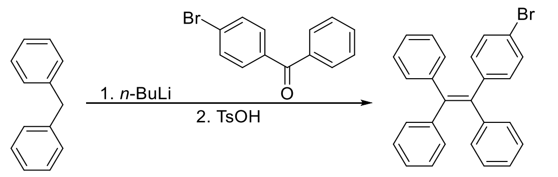

Synthesis of (2-(4-bromophenyl)ethene-1,1,2-triyl)tribenzene (1)

To a 250 mL of two-necked round-bottom flask containing diphenylmethane (3.36 g, 20 mmol), 100 mL of tetrahydrofuran (THF) was injected under argon atmosphere, followed by cooling down to 0 °C with an ice-water bath. After 15 min, *n*-BuLi (20 mmol, 2.0 M in hexane, 10 mL) was added dropwise, and the solution was stirred at 0 ^{o}C for 1 h. Afterwards, (4-bromophenyl)(phenyl)methanone (5.2 g, 20 mmol) was added, and the reaction mixture was warmed to room temperature and stirred overnight. Aqueous NH_4Cl solution was added to quench the reaction. The mixture was extracted with dichloromethane three times. The organic layers were combined, washed with saturated brine solution, and dried with anhydrous $MgSO_4$. The solvent was evaporated under reduced pressure, and the resulting product was used without further purification. The crude alcohol and *p*-toluenesulphonic acid (0.9 g, 5 mmol) were added

into a 500 mL of two-necked round-bottom flask. Then, 200 mL of toluene was added into the flask under argon atmosphere, and the mixture was heated to reflux and stirred overnight. Then aqueous $NaHCO_3$ solution was added, and the organic layer was combined and dried with $MgSO_4$. The solvent was evaporated, and the crude product was purified by silica gel chromatography (eluent: hexane) to afford (2-(4-bromophenyl)ethene-1,1,2-triyl)tribenzene as a white solid (85% yield). ^{1}H NMR (400 MHz, $CDCl_3$): δ 7.23–7.19 (m, 2H), 7.15–7.06 (m, 9H), 7.05–6.97 (m, 6H), 6.92–6.86 (m, 2H). ^{13}C NMR (100 MHz, $CDCl_3$): δ 143.44, 143.36, 143.25, 142.73, 141.65, 139.69, 133.01, 131.32, 131.27, 131.25, 130.88, 127.91, 127.81, 127.71, 126.73, 126.68, 126.62, 120.48.

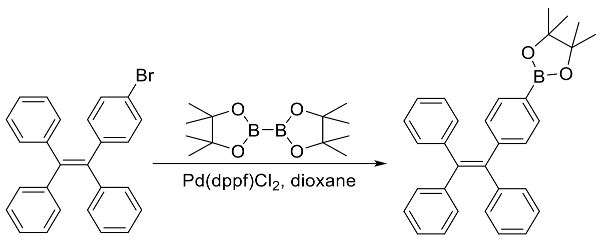

Synthesis of 4,4,5,5-tetramethyl-2-(4-(1,2,2-triphenylvinyl)phenyl)-1,3,2-dioxaborolane (2)

(2-(4-Bromophenyl)ethene-1,1,2-triyl)tribenzene (4.1 g, 10 mmol), 1,1'-bis(diphenylphosphino)ferrocene-palladium(II)dichloride dichloromethane complex (0.245 g, 0.3 mmol), KOAc (3.0 g, 30 mmol), and bis(pinacolato)diboron (3.81 g, 15 mmol) were added into a 100 mL of two-necked round-bottom flask. 1,4-Dioxane (40 mL) was added under argon atmosphere, and the mixture was heated to reflux and stirred for 24 h. Afterwards, water was added, and the mixture was extracted with dichloromethane three times. The organic phase was combined, dried with $MgSO_4$, and the solvent was evaporated under reduced pressure. The crude product was purified by silica gel chromatography (eluent: dichloromethane/hexane 1/2) to afford 4,4,5,5-tetramethyl-2-(4-(1,2,2-triphenylvinyl)phenyl)-1,3,2-dioxaborolane as a white solid (76% yield). ^{1}H NMR (400 MHz, $CDCl_3$): δ 7.54 (d, J = 7.9 Hz, 2H), 7.08 (q, J = 3.4 Hz, 9H), 7.02 (td, J = 6.8, 6.0, 3.0 Hz, 8H), 1.31 (s, 12H). ^{13}C NMR (100 MHz, $CDCl_3$): δ 146.78, 143.71, 143.61, 143.53, 141.40, 140.87, 134.11, 131.36, 131.33, 131.32, 130.71, 128.95, 128.47, 127.74, 127.65, 126.54, 126.47, 126.45, 83.70, 25.05.

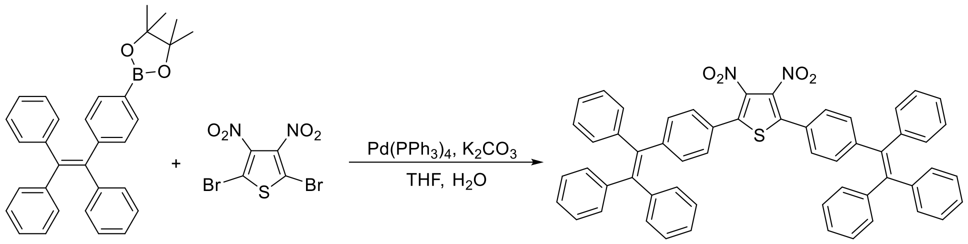

Synthesis of 3,4-dinitro-2,5-bis(4-(1,2,2-triphenylvinyl)phenyl)thiophene (4)

4,4,5,5-Tetramethyl-2-(4-(1,2,2-triphenylvinyl)phenyl)-1,3,2-dioxaborolane (2.29 g, 5 mmol), 2,5-dibromo-3,4-dinitrothiophene (0.67 g, 2 mmol), $Pd(PPh_3)_4$ (60 mg, 0.05 mmol), and K_2CO_3 (2.07 g, 15 mmol) were added into a 100 mL of two-necked round-bottom flask. The flask was vacuumed and purged with argon for three times. Then tetrahydrofuran (40 mL) and water (10 mL) were added and the mixture was heated to reflux and stirred for 24 h in the absence of light. Afterwards, water was added, and the mixture was extracted with dichloromethane three times. The organic phase was combined, and dried with $MgSO_4$. After removal of the solvent under reduced pressure, the residue was purified by silica gel chromatography (eluent: dichloromethane/hexane 1/5) to afford 3,4-dinitro-2,5-bis(4-(1,2,2-triphenylvinyl)phenyl)thiophene as a yellow-orange solid (82% yield). ^{1}H NMR (400 MHz, $CDCl_3$): δ 7.21 (d, J = 8.2 Hz, 4H), 7.17–7.06 (m, 22H), 7.04 (d, J = 7.6 Hz, 12H). ^{13}C NMR (100 MHz, $CDCl_3$): δ 146.67, 143.19, 143.05, 142.96, 142.78, 140.35, 139.53, 136.61, 132.38, 132.05, 131.36, 131.31, 131.29, 131.25, 128.24, 127.95, 127.75, 127.56, 127.07, 127.04, 126.87, 126.84, 126.82, 125.91. HRMS (MALDI-TOF, *m*/*z*): $[M]^+$ calcd for $C_{56}H_{38}N_2O_4S$, 834.2552; found, 834.2534.

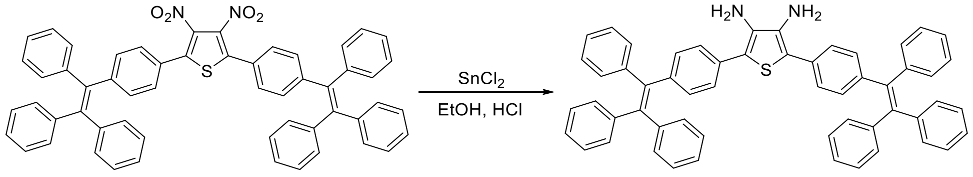

Synthesis of 2,5-bis(4-(1,2,2-triphenylvinyl)phenyl)thiophene-3,4-diamine (5)

3,4-Dinitro-2,5-bis(4-(1,2,2-triphenylvinyl)phenyl)thiophene (0.835 g, 1 mmol) was added to the mixture ethanol (20 mL) and concentrated HCl (15 mL) in a 100 mL of two-necked round-bottom flask. Then $SnCl_2 \cdot 2H_2O$ (4.5 g, 20 mmol) was added to the suspension, which was refluxed under argon atmosphere for 20 h. After cooling down to 0 ^oC, 50 mL of 25 wt% KOH aqueous solution was added, and the mixture was extracted with dichloromethane three times.

The organic phase was combined, and dried with $MgSO_4$. After removal of the solvent under reduced pressure, the crude product of 2,5-bis(4-(1,2,2-triphenylvinyl)phenyl)thiophene-3,4-diamine was used without further purification.

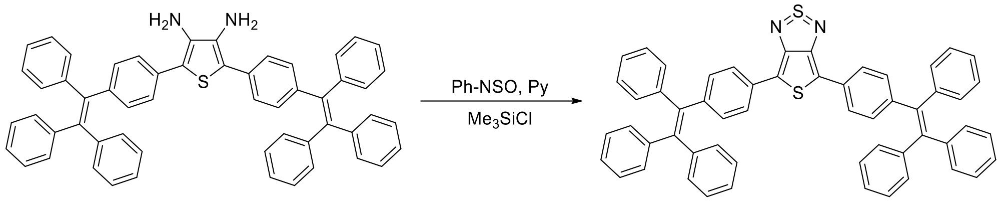

Synthesis of 4,8-bis[4-(1,2,2-triphenylvinyl)phenyl]thieno[3,4-b][1,2,5]thiadiazole (TPE-TT)

2,5-Bis(4-(1,2,2-triphenylvinyl)phenyl)thiophene-3,4-diamine (1 mmol) was dissolved in dry pyridine (35 mL) under argon atmosphere, which was heated to 80 °C. And *N*-thionylaniline (0.28 g, 2 mmol) and Me_3SiCl (0.45 g, 3 mmol) was added to the solution, and the reaction was continued at 80 °C overnight. Most of pyridine was evaporated under reduced pressure and the residue was purified by silica gel chromatography (eluent: dichloromethane/hexane 1/3) to afford 4,8-bis[4-(1,2,2-triphenylvinyl)phenyl]thieno[3,4-*b*][1,2,5]thiadiazole (TPE-TT) as a dark blue solid (62% yield). ^1H NMR (400 MHz, $CDCl_3$): δ 7.84 (d, J = 8.5 Hz, 4H), 7.15–7.05 (m, 30H), 7.03 (dd, J = 6.7, 3.0 Hz, 4H). ^{13}C NMR (100 MHz, $CDCl_3$): δ 157.53, 143.68, 143.64, 143.51, 143.12, 141.51, 140.35, 132.18, 131.46, 131.36, 130.80, 127.89, 127.77, 127.65, 126.71, 126.60, 126.52, 124.95, 118.82. HRMS (MALDI-TOF, *m/z*): $[M]^+$ calcd. for $C_{56}H_{38}N_2S_2$, 802.2476; found, 802.2455.

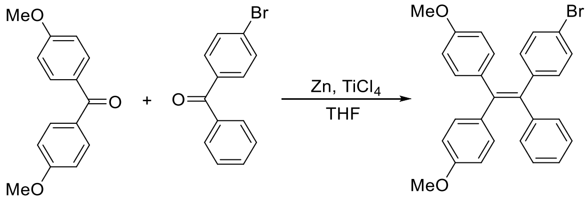

Synthesis of 4,4'-(2-(4-bromophenyl)-2-phenylethene-1,1-diyl)bis(methoxybenzene) (6)

Bis(4-methoxyphenyl)methanone (7.26 g, 30 mmol), (4-bromophenyl)(phenyl)methanone (7.83 g, 30 mmol), and zinc powder (7.8 g, 120 mmol) were added into a 250 mL of two-necked round-bottom flask. The flask was then vacuumed and purged with argon three times, and anhydrous THF (150 mL) was added. The mixture was then cooled with ice-water to 0 °C, and $TiCl_4$ (9.9 mL, 90 mmol) was added dropwise. Then the mixture was heated to reflux, and

stirred overnight. After cooling down to 0 °C, the reaction was quenched by the adding aqueous HCl (1 M) and stirred for 2 h. The mixture was washed with dichloromethane three times. The organic phase was combined, dried with $MgSO_4$, and the solvent was evaporated under reduced pressure. The crude product was purified by silica gel chromatography (eluent: dichloromethane/hexane 1/4) to afford 4,4'-(2-(4-bromophenyl)-2-phenylethene-1,1-diyl)bis(methoxybenzene) as a white solid (40% yield). ^{1}H NMR (400 MHz, $CDCl_3$): δ 7.22 (d, J = 7.2 Hz, 2H), 7.11 (d, J = 6.5 Hz, 3H), 7.00 (d, J = 5.6 Hz, 2H), 6.97–6.85 (m, 6H), 6.65 (dd, J = 13.8, 8.1 Hz, 4H), 3.75 (d, J = 11.0 Hz, 6H). ^{13}C NMR (100 MHz, $CDCl_3$): δ 158.31, 158.22, 143.81, 143.32, 140.81, 137.93, 136.04, 135.95, 133.05, 132.56, 132.53, 131.35, 130.87, 127.82, 127.68, 126.32, 120.03, 113.23, 113.05, 55.13, 55,10.

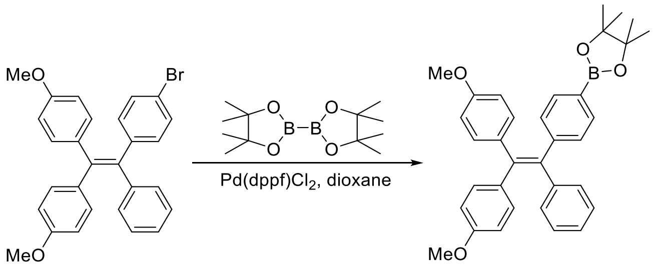

Synthesis of 2-(4-(2,2-bis(4-methoxyphenyl)-1-phenylvinyl)phenyl)-4,4,5,5-tetramethyl-1,3,2-dioxaborolane (7)

4,4'-(2-(4-Bromophenyl)-2-phenylethene-1,1-diyl)bis(methoxybenzene) (5.18 g, 10 mmol), 1,1'-bis(diphenylphosphino)ferrocene-palladium(II)dichloride dichloromethane complex (0.245 g, 0.3 mmol), KOAc (3.0 g, 30 mmol), and bis(pinacolato)diboron (3.81 g, 15 mmol) were added into a 100 mL of two-necked round-bottom flask. 1,4-Dioxane (40 mL) was added under argon atmosphere, and the mixture was heated to reflux and stirred for 24 h. Afterwards, water was added, and the mixture was extracted with dichloromethane three times. The organic phase was combined, dried with $MgSO_4$, and the solvent was evaporated under reduced pressure. The crude product was purified by silica gel chromatography (eluent: dichloromethane/hexane 1/2) to afford 2-(4-(2,2-bis(4-methoxyphenyl)-1-phenylvinyl)phenyl)-4,4,5,5-tetramethyl-1,3,2-dioxaborolane as a white solid (78% yield). ^{1}H NMR (400 MHz, $CDCl_3$): δ 7.54 (d, J = 7.7 Hz, 2H), 7.08 (d, J = 7.1 Hz, 3H), 7.05–6.97 (m, 4H), 6.96–6.89 (m, 4H), 6.63 (d, J = 7.9 Hz, 4H), 3.74 (d, J = 2.6 Hz, 6H), 1.26 (s, 12H). ^{13}C NMR (100 MHz, $CDCl_3$): δ 158.16, 158.13, 147.42, 144.14, 140.60, 139.14, 136.31, 136.20, 134.11, 132.59, 131.41, 130.77, 127.67, 126.11, 113.09, 113.00, 83.66, 55.07, 55.05, 25.04.

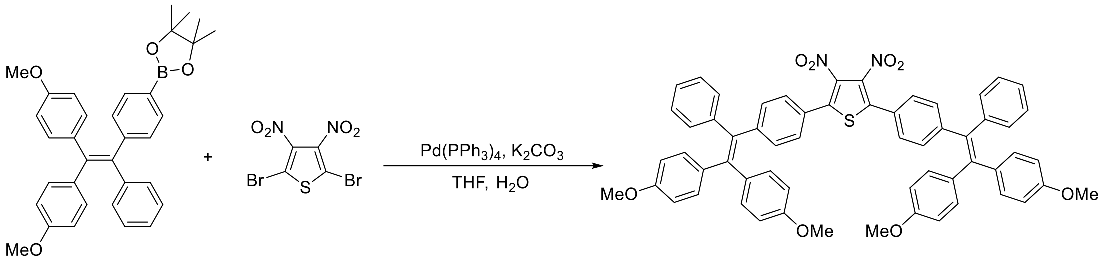

Synthesis of 2,5-bis(4-(2,2-bis(4-methoxyphenyl)-1-phenylvinyl)phenyl)-3,4-dinitrothiophene (8)

2-(4-(2,2-Bis(4-methoxyphenyl)-1-phenylvinyl)phenyl)-4,4,5,5-tetramethyl-1,3,2-dioxaborolane (2.59 g, 5 mmol), 2,5-dibromo-3,4-dinitrothiophene (0.67 g, 2 mmol), $Pd(PPh_3)_4$ (60 mg, 0.05 mmol), and K_2CO_3 (2.07 g, 15 mmol) were added into a 100 mL of two-necked round-bottom flask. The flask was vacuumed and purged with argon three times. Then THF (40 mL) and water (10 mL) were added and the mixture was heated to reflux and stirred for 24 h in the absence of light. Afterwards, water was added, and the mixture was extracted with dichloromethane three times. The organic phase was combined, and dried with $MgSO_4$. After removal of the solvent under reduced pressure, the residue was purified by silica gel chromatography (eluent: dichloromethane/hexane 1/3) to afford 2,5-bis(4-(2,2-bis(4-methoxyphenyl)-1-phenylvinyl)phenyl)-3,4-dinitrothiophene as an orange solid (80% yield). ^{1}H NMR (400 MHz, $CDCl_3$): δ 7.21 (d, J = 8.5 Hz, 4H), 7.17–7.08 (m, 10H), 7.04 (dd, J = 7.7, 1.8 Hz, 4H), 6.97–6.90 (m, 8H), 6.65 (dd, J = 11.5, 8.8 Hz, 8H), 3.75 (d, J = 7.1 Hz, 12H). ^{13}C NMR (100 MHz, $CDCl_3$): δ 158.57, 158.37, 147.34, 143.54, 142.01, 140.41, 137.73, 136.55, 135.76, 135.61, 132.68, 132.61, 132.09, 131.42, 128.24, 127.98, 126.50, 125.50, 113.28, 113.07, 55.19, 55.12. HRMS (MALDI-TOF, *m*/*z*): $[M]^+$ calcd. for $C_{60}H_{46}N_2O_8S$, 954.2975; found, 954.2989.

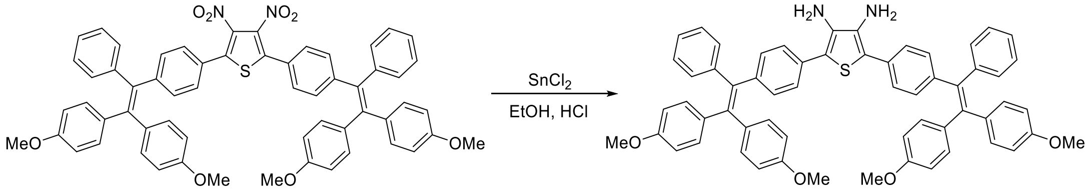

Synthesis of 2,5-bis(4-(2,2-bis(4-methoxyphenyl)-1-phenylvinyl)phenyl)thiophene-3,4-diamine (9)

2,5-Bis(4-(2,2-bis(4-methoxyphenyl)-1-phenylvinyl)phenyl)-3,4-dinitrothiophene (0.954 g, 1 mmol) was added to the mixture ethanol (20 mL) and concentrated HCl (15 mL) in a 100 mL of two-necked round-bottom flask. Then $SnCl_2·2H_2O$ (4.5 g, 20 mmol) was added to the suspension, which was refluxed under argon atmosphere for 20 h. After cooling down to 0 ^oC, 50 mL of 25 wt% KOH aqueous solution was added, and the mixture was extracted with dichloromethane three times. The organic phase was combined, and dried with $MgSO_4$. After removal of the solvent under reduced pressure, the crude product of 2,5-bis(4-(2,2-bis(4-methoxyphenyl)-1-phenylvinyl)phenyl)thiophene-3,4-diamine was used without further purification.

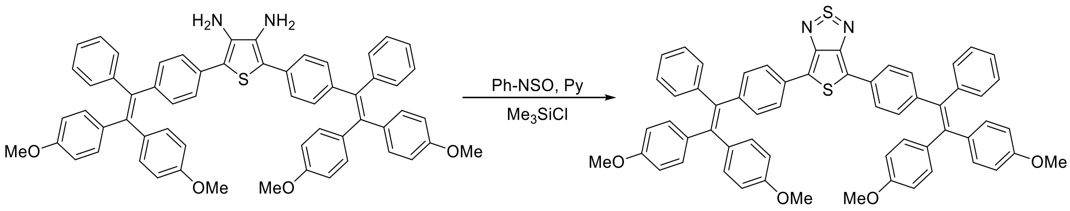

Synthesis of 4,8-bis{4-[2,2-bis(4-methoxyphenyl)-1-phenylvinyl]phenyl}thieno[3,4-b][1,2,5]thiadiazole (MTPE-TT)

2,5-Bis(4-(2,2-bis(4-methoxyphenyl)-1-phenylvinyl)phenyl)thiophene-3,4-diamine (1 mmol) was dissolved in dry pyridine (35 mL) under argon atmosphere, which was heated to 80 °C. And *N*-thionylaniline (0.28 g, 2 mmol) and Me_3SiCl (0.45 g, 3 mmol) was added to the solution, and the reaction was continued at 80 °C overnight. Most of pyridine was evaporated under reduced pressure and the residue was purified by silica gel chromatography (eluent: dichloromethane/hexane 1/3) to afford 4,8-bis{4-[2,2-bis(4-methoxyphenyl)-1-phenylvinyl]phenyl}thieno[3,4-*b*][1,2,5]thiadiazole (MTPE-TT) as a dark blue solid (67% yield). ^{1}H NMR (400 MHz, $CDCl_3$): δ 7.85 (d, J = 8.4 Hz, 4H), 7.16–7.04 (m, 14H), 7.01 (d, J = 8.7 Hz, 4H), 6.94 (d, J = 8.7 Hz, 4H), 6.65 (dd, J = 14.1, 8.8 Hz, 8H), 3.74 (d, J = 1.2 Hz, 12H). ^{13}C NMR (100 MHz, $CDCl_3$): δ 158.31, 158.16, 157.49, 144.08, 143.73, 140.72, 138.63, 136.30, 136.28, 132.65, 132.21, 131.52, 130.49, 127.80, 126.26, 124.96, 118.77, 113.25, 113.01, 55.10. HRMS (MALDI-TOF, *m*/*z*): $[M]^+$ calcd. for $C_{60}H_{46}N_2O_4S_2$, 922.2899; found, 922.2884.

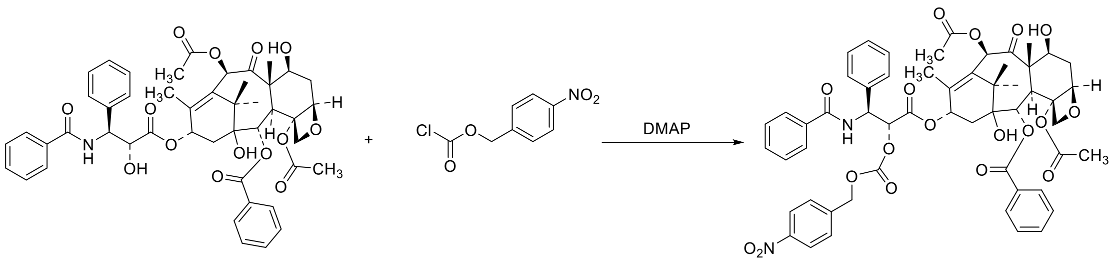

Synthesis of (2aR,4S,4aS,6R,9S,12S,12aR,12bS)-9-(((2R,3S)-3-benzamido-2-((((4-nitrobenzyl)oxy)carbonyl)oxy)-3-phenylpropanoyl)oxy)-12-(benzoyloxy)-4,11-dihydroxy-4a,8,13,13-tetramethyl-5-oxo-3,4,4a,5,6,9,10,11,12,12a-decahydro-1H-7,11-methanocyclodeca[3,4]benzo[1,2-b]oxete-6,12b(2aH)-diyl diacetate (PTX-NB)

PTX (150 mg, 0.18 mmol) was dissolved in 15 mL of dry CH_2Cl_2, and the solution was cooled to 0 ^oC. Catalytic amount 4-dimethylaminopyridine (DMAP, 25.8 mg, 0.21 mmol) was added, and 4-nitrobenzyl chloroformate (45.5 mg, 0.21 mmol) solution in dry CH_2Cl_2 was added dropwise. The mixture was stirred at room temperature overnight. Afterwards, the mixture was extracted with CH_2Cl_2 three times. The organic layer was separated, dried with anhydrous $MgSO_4$, and evaporated under reduced pressure. The crude product was purified by silica gel chromatography (eluent: ethyl acetate/hexane 1/2) to afford PTX-NB as a white solid (70% yield). ^1H NMR (400 MHz, $CDCl_3$): δ 8.24–8.19 (m, 2H), 8.17–8.11 (m, 2H), 7.76–7.69 (m, 2H), 7.64–7.57 (m, 1H), 7.54–7.46 (m, 5H), 7.45–7.36 (m, 7H), 6.91 (d, J = 9.4 Hz, 1H), 6.29 (d, J = 4.1 Hz, 2H), 6.02 (dd, J = 9.4, 2.7 Hz, 1H), 5.69 (d, J = 7.0 Hz, 1H), 5.46 (d, J = 2.8 Hz, 1H), 5.32–5.20 (m, 3H), 4.97 (dd, J = 9.6, 2.3 Hz, 1H), 4.43 (dd, J = 10.9, 6.6 Hz, 1H), 4.32 (d, J = 8.3 Hz, 1H), 4.21 (dd, J = 8.5, 1.0 Hz, 1H), 3.81 (d, J = 7.0 Hz, 1H), 3.41–3.35 (m, 2H), 2.84 (d, J = 0.8 Hz, 3H), 2.46 (s, 3H), 2.23 (s, 4H), 1.91 (s, 3H), 1.69 (s, 3H), 1.14 (s, 3H), 0.91–0.84 (m, 2H).

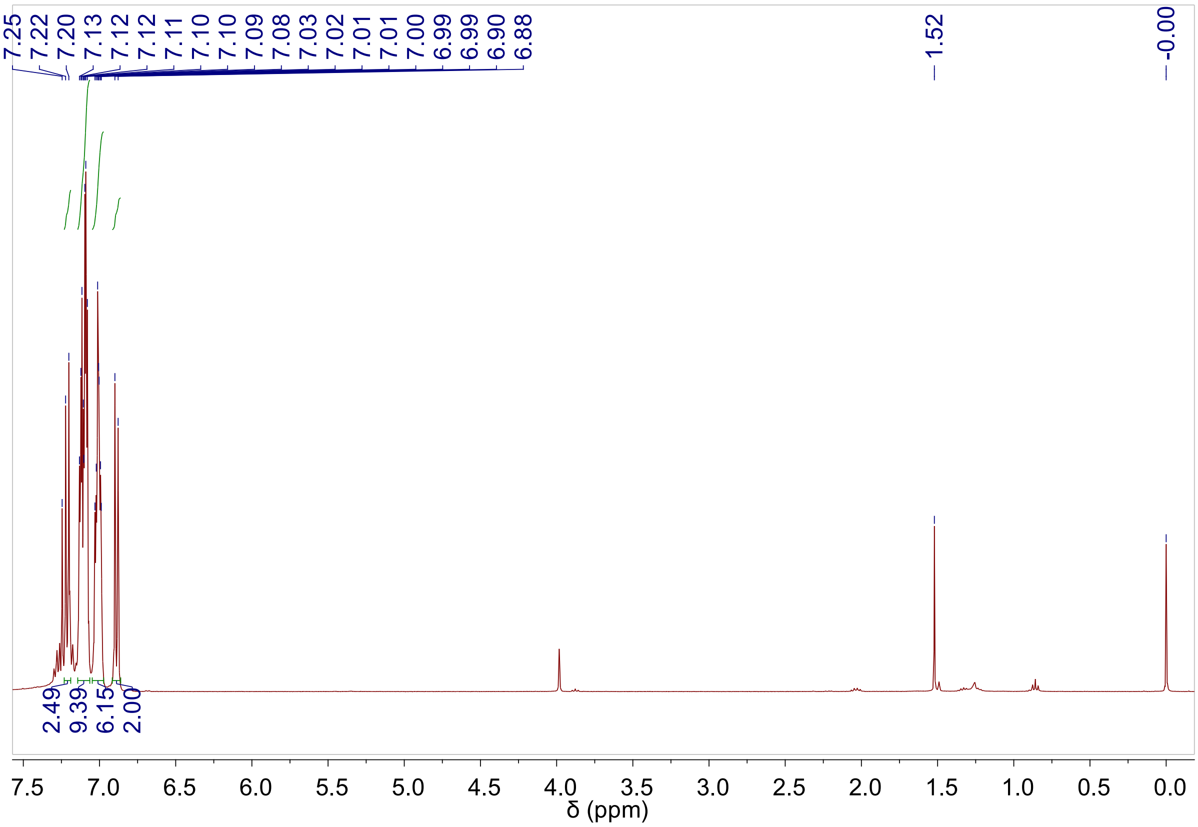

Supplementary Fig. 3. ^{1}H NMR spectrum of (2-(4-bromophenyl)ethene-1,1,2-triyl)tribenzene in $CDCl_3$ at 298 K.

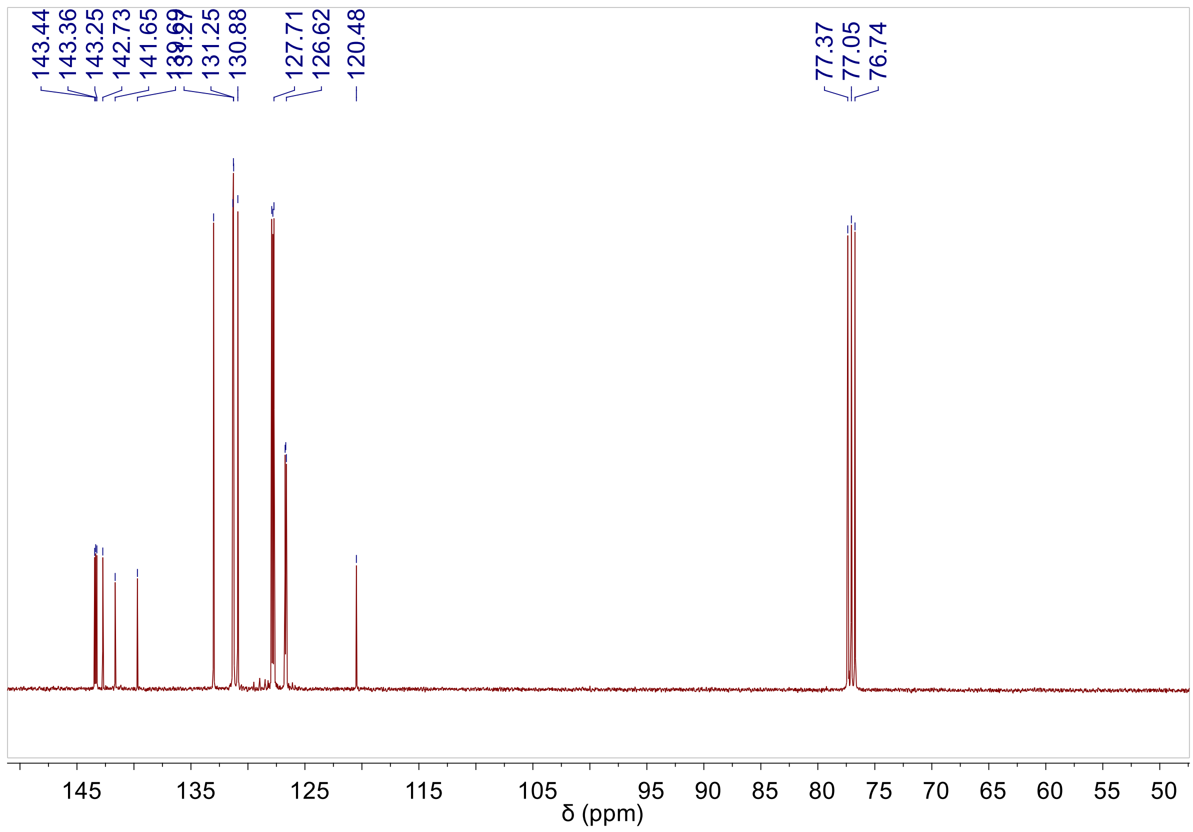

Supplementary Fig. 4. ^{13}C NMR spectrum of (2-(4-bromophenyl)ethene-1,1,2-triyl)tribenzene in $CDCl_3$ at 298 K.

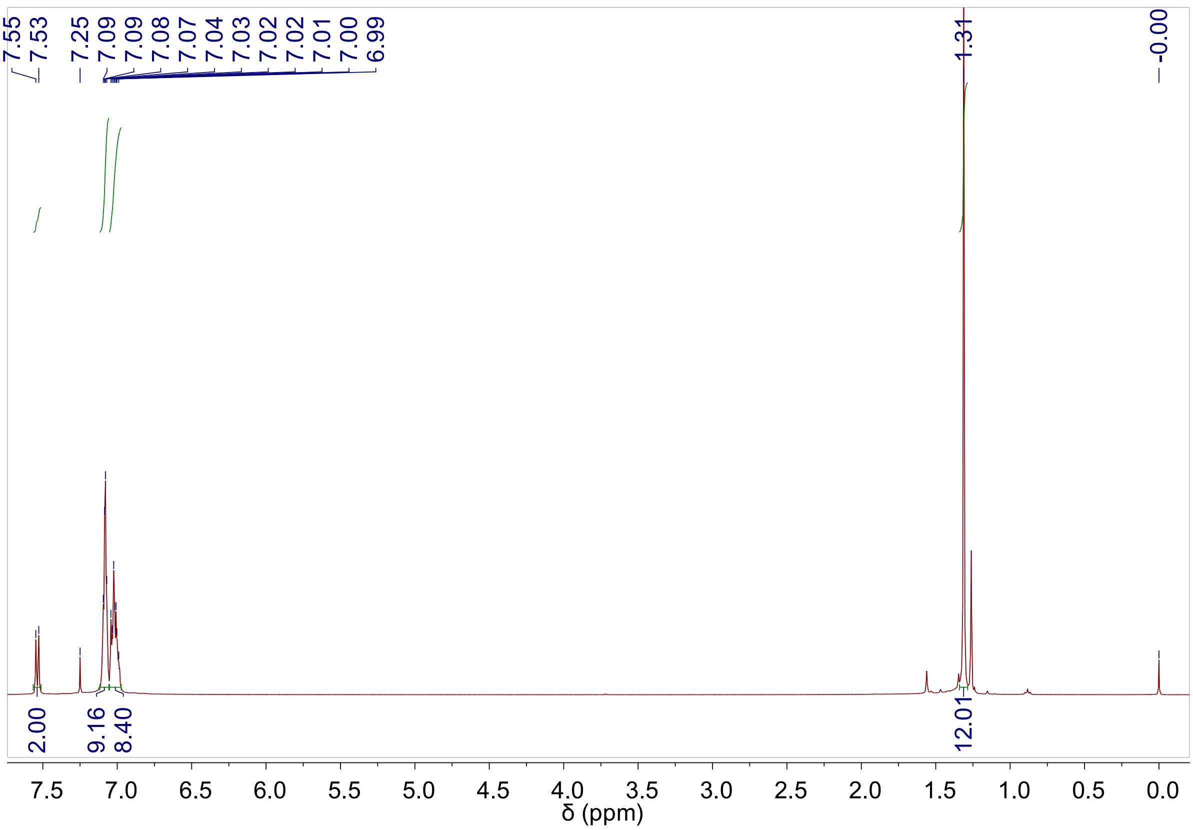

Supplementary Fig. 5. ^{1}H NMR spectrum of 4,4,5,5-tetramethyl-2-(4-(1,2,2-triphenylvinyl)phenyl)-1,3,2-dioxaborolane in $CDCl_3$ at 298 K.

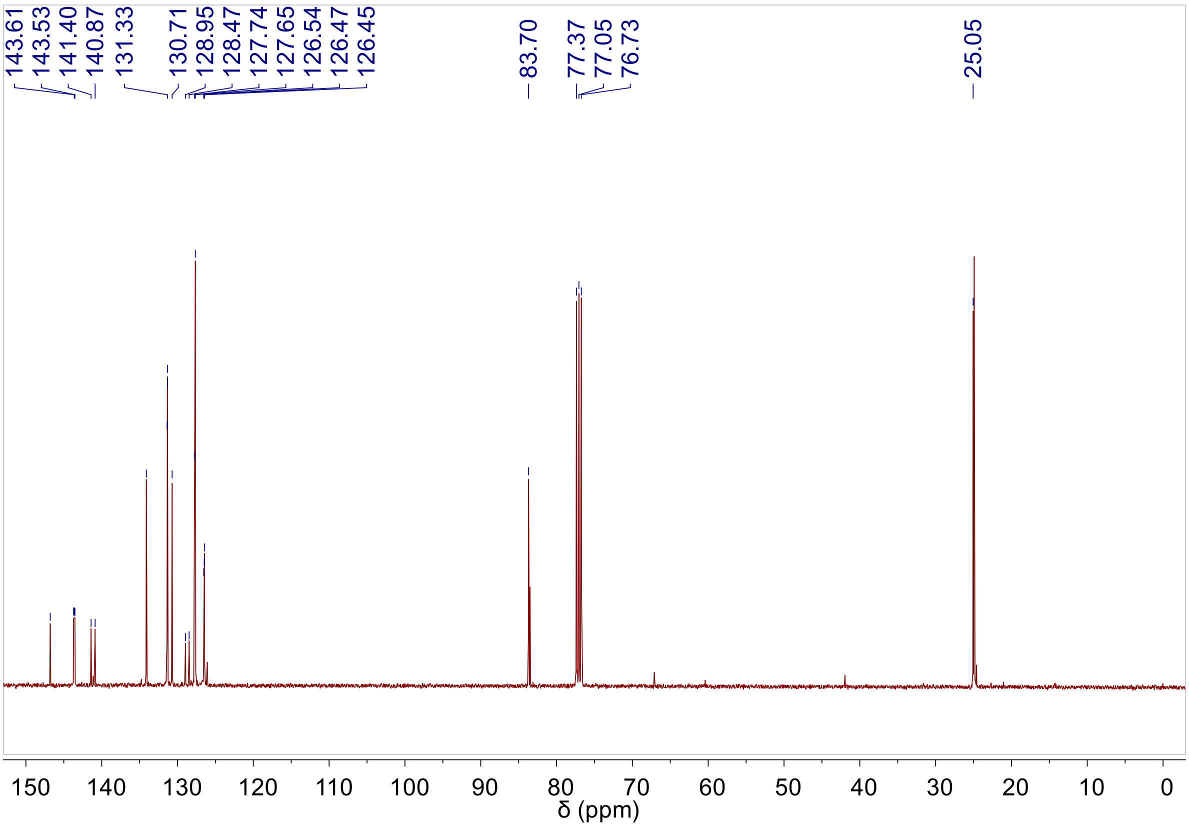

Supplementary Fig. 6. ^{13}C NMR spectrum of 4,4,5,5-tetramethyl-2-(4-(1,2,2-triphenylvinyl)phenyl)-1,3,2-dioxaborolane in $CDCl_3$ at 298 K.

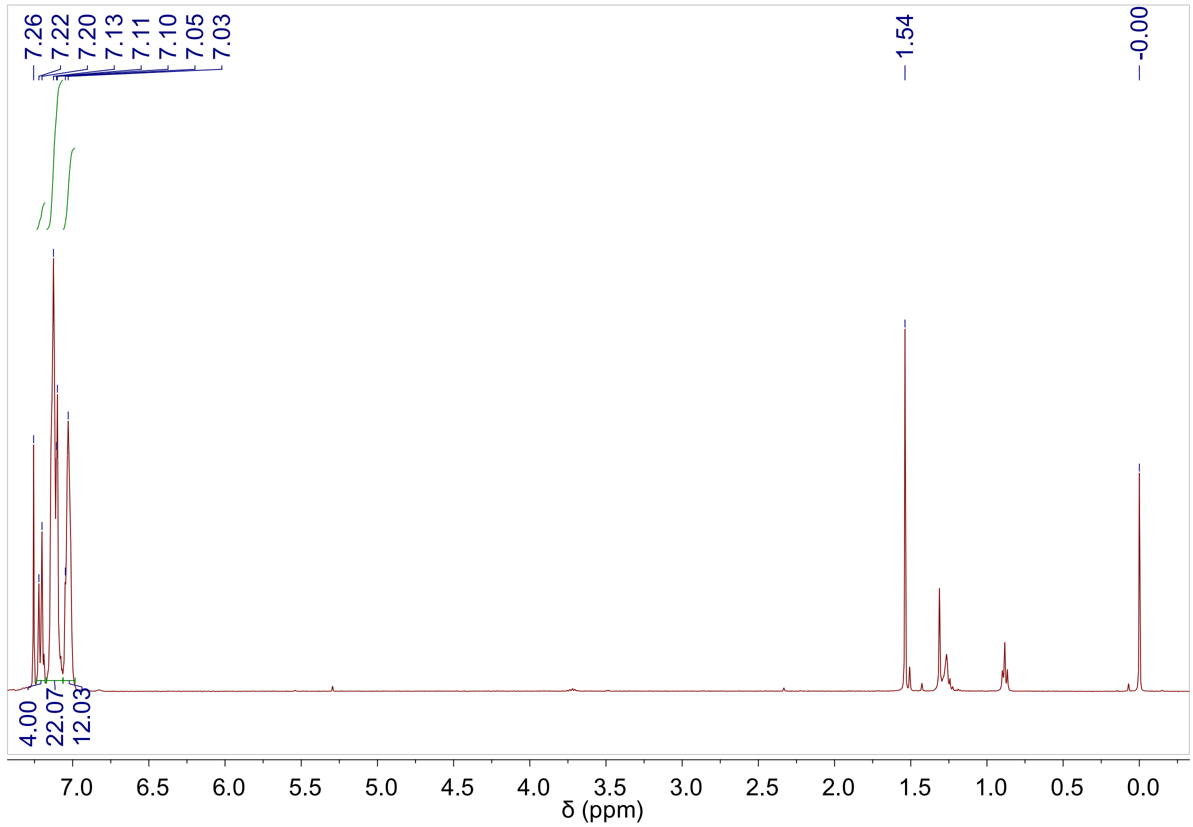

Supplementary Fig. 7. ^{1}H NMR spectrum of 3,4-dinitro-2,5-bis(4-(1,2,2-triphenylvinyl)phenyl)thiophene in $CDCl_3$ at 298 K.

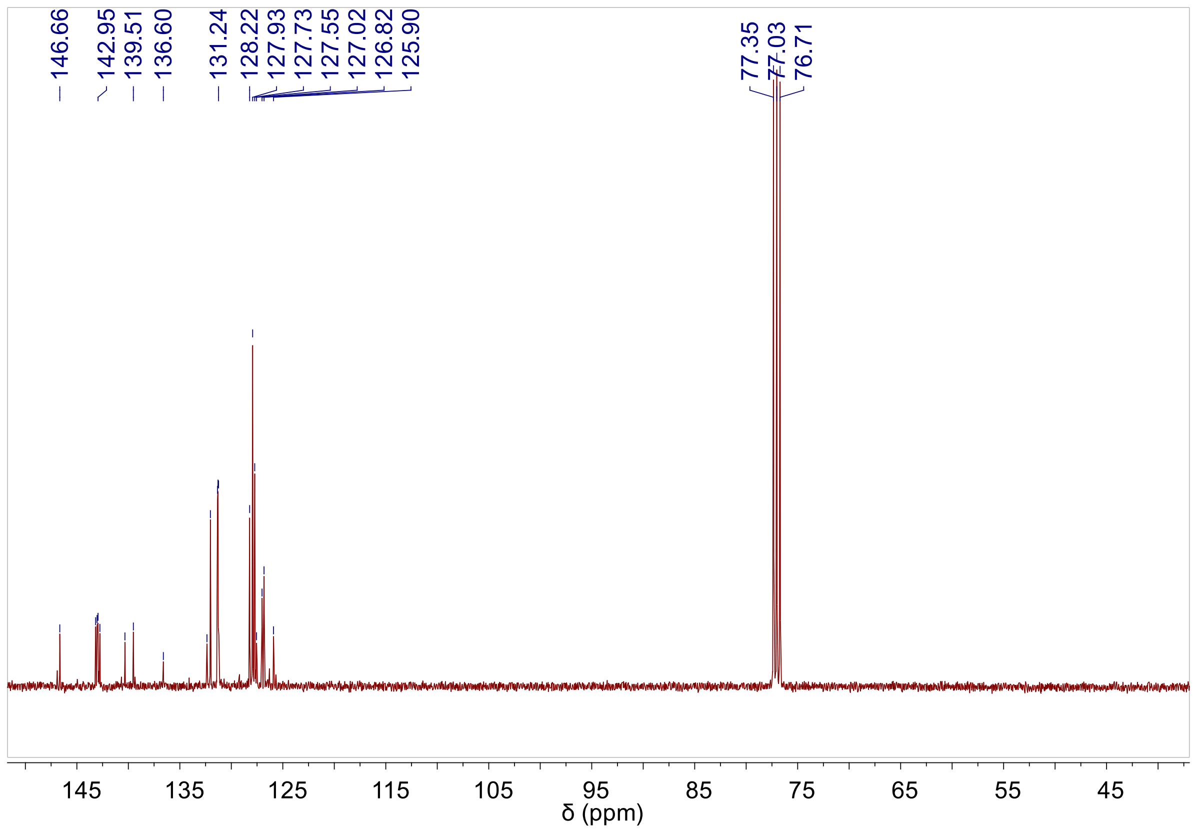

Supplementary Fig. 8. ^{13}C NMR spectrum of 3,4-dinitro-2,5-bis(4-(1,2,2-triphenylvinyl)phenyl)thiophene in $CDCl_3$ at 298 K.

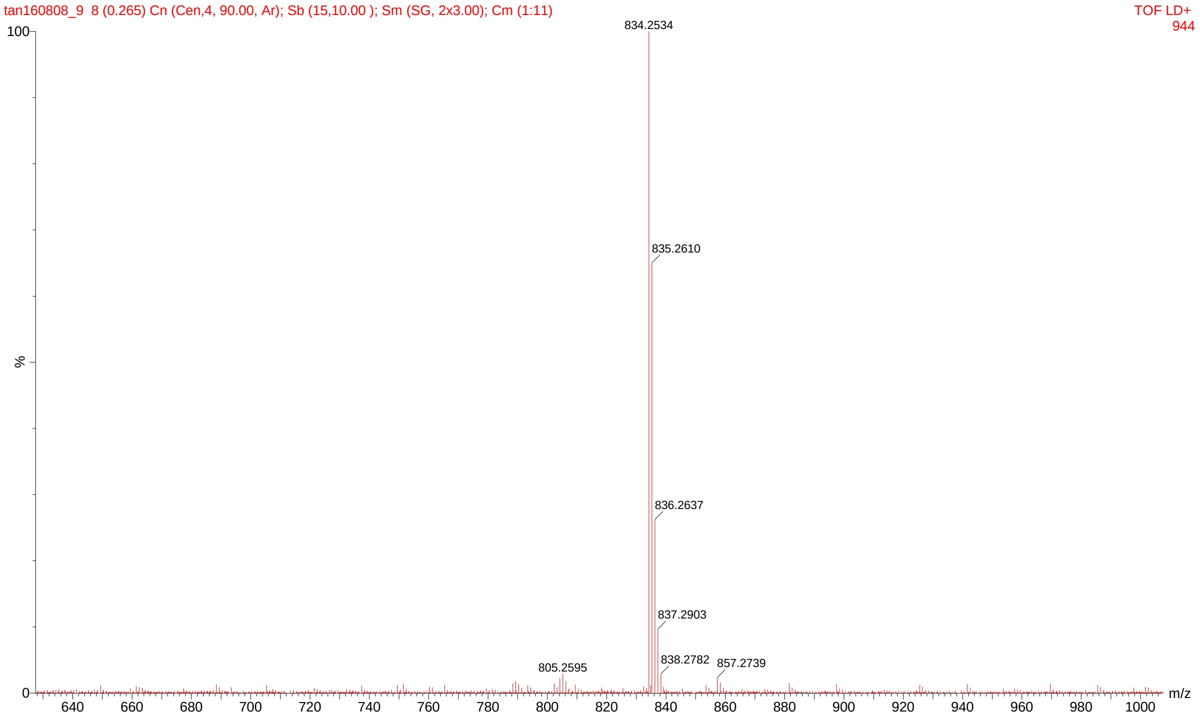

Supplementary Fig. 9. HRMS of 3,4-dinitro-2,5-bis(4-(1,2,2-triphenylvinyl)phenyl)thiophene.

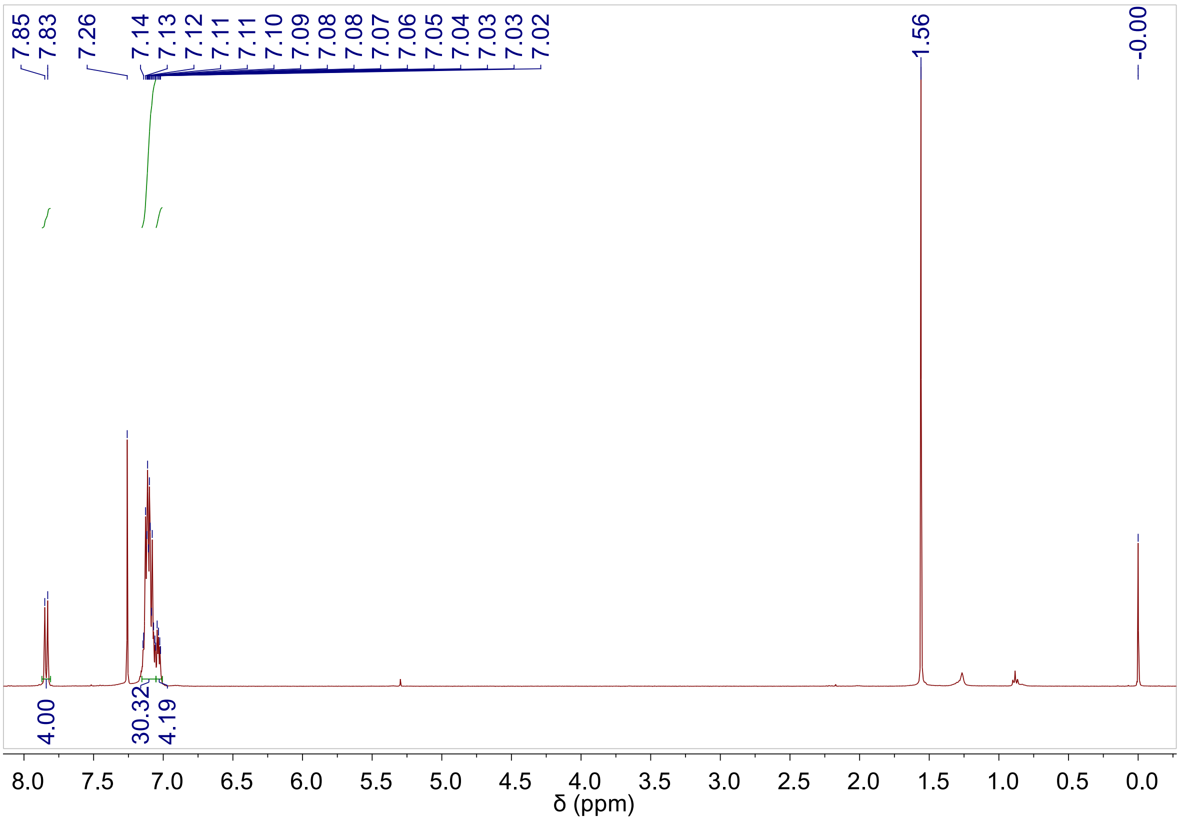

Supplementary Fig. 10. ^{1}H NMR spectrum of TPE-TT in $CDCl_3$ at 298 K.

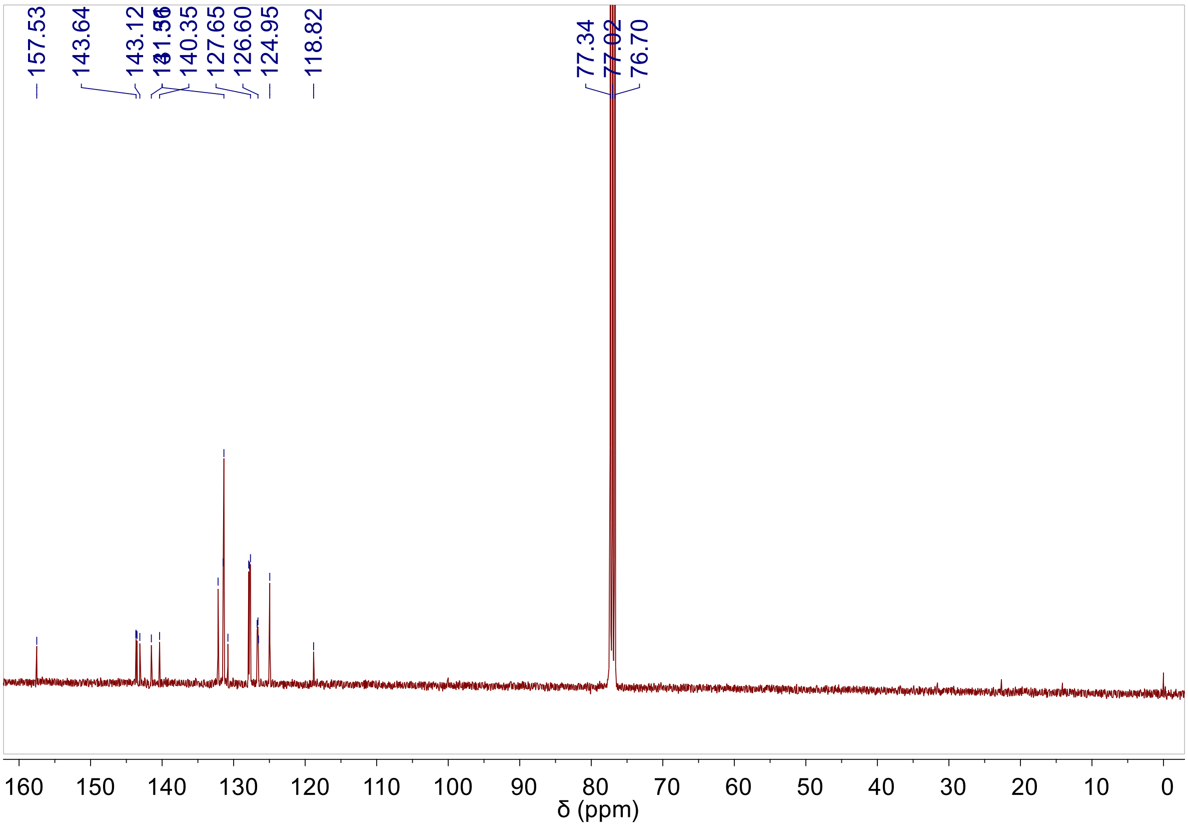

Supplementary Fig. 11. ^{13}C NMR spectrum of TPE-TT in $CDCl_3$ at 298 K.

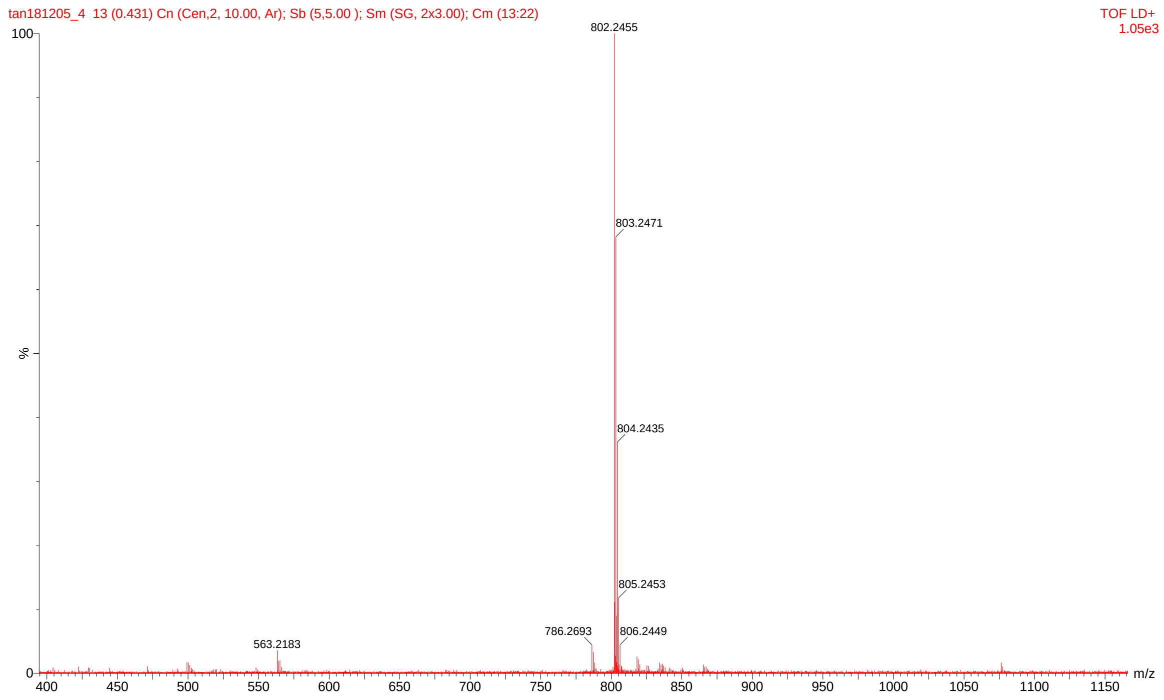

Supplementary Fig. 12. HRMS of TPE-TT.

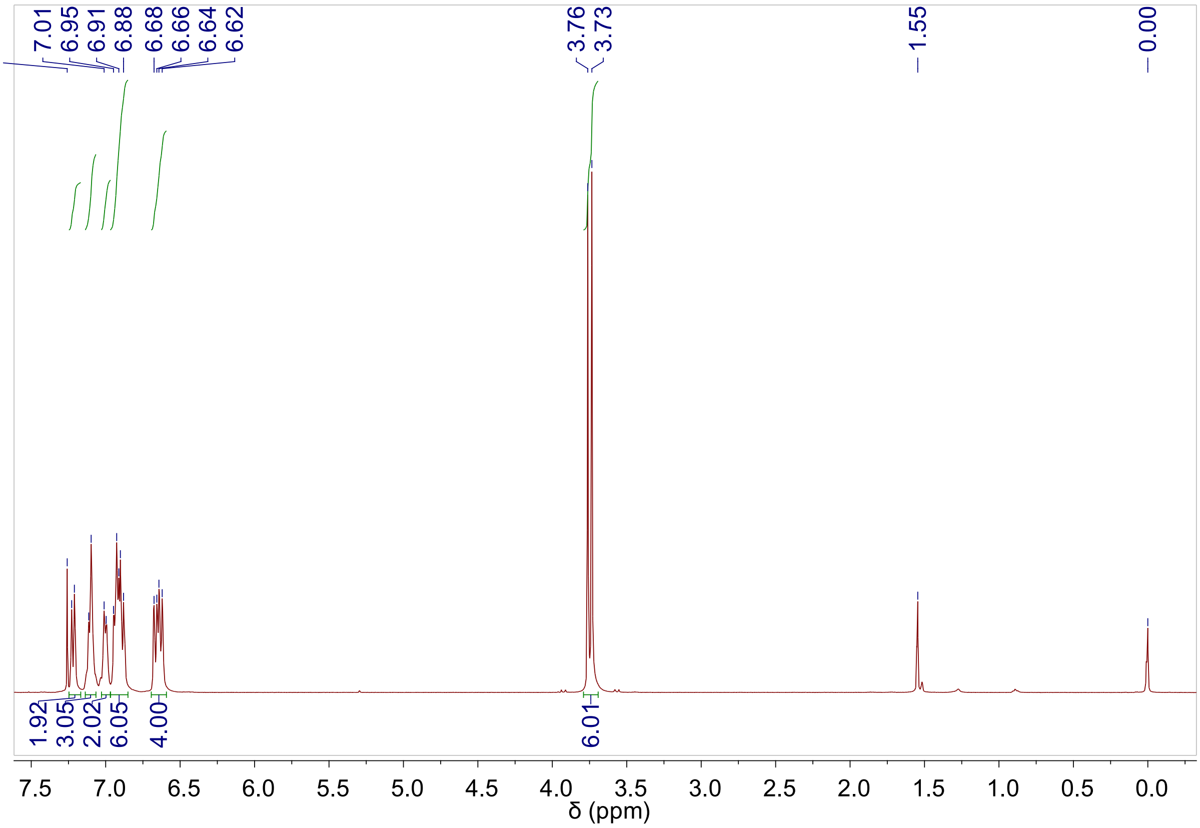

Supplementary Fig. 13. ^{1}H NMR spectrum of 4,4'-(2-(4-bromophenyl)-2-phenylethene-1,1-diyl)bis(methoxybenzene) in $CDCl_3$ at 298 K.

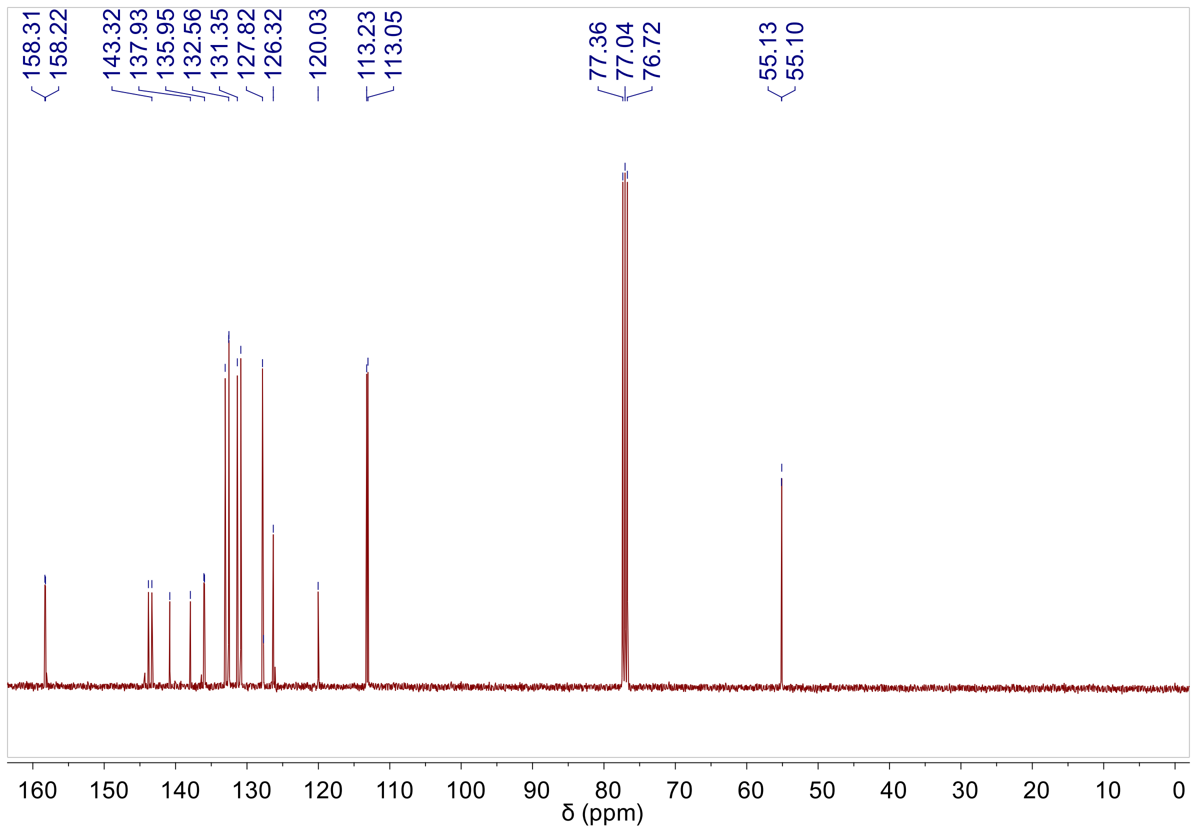

Supplementary Fig. 14. ^{13}C NMR spectrum of 4,4'-(2-(4-bromophenyl)-2-phenylethene-1,1-diyl)bis(methoxybenzene) in $CDCl_3$ at 298 K.

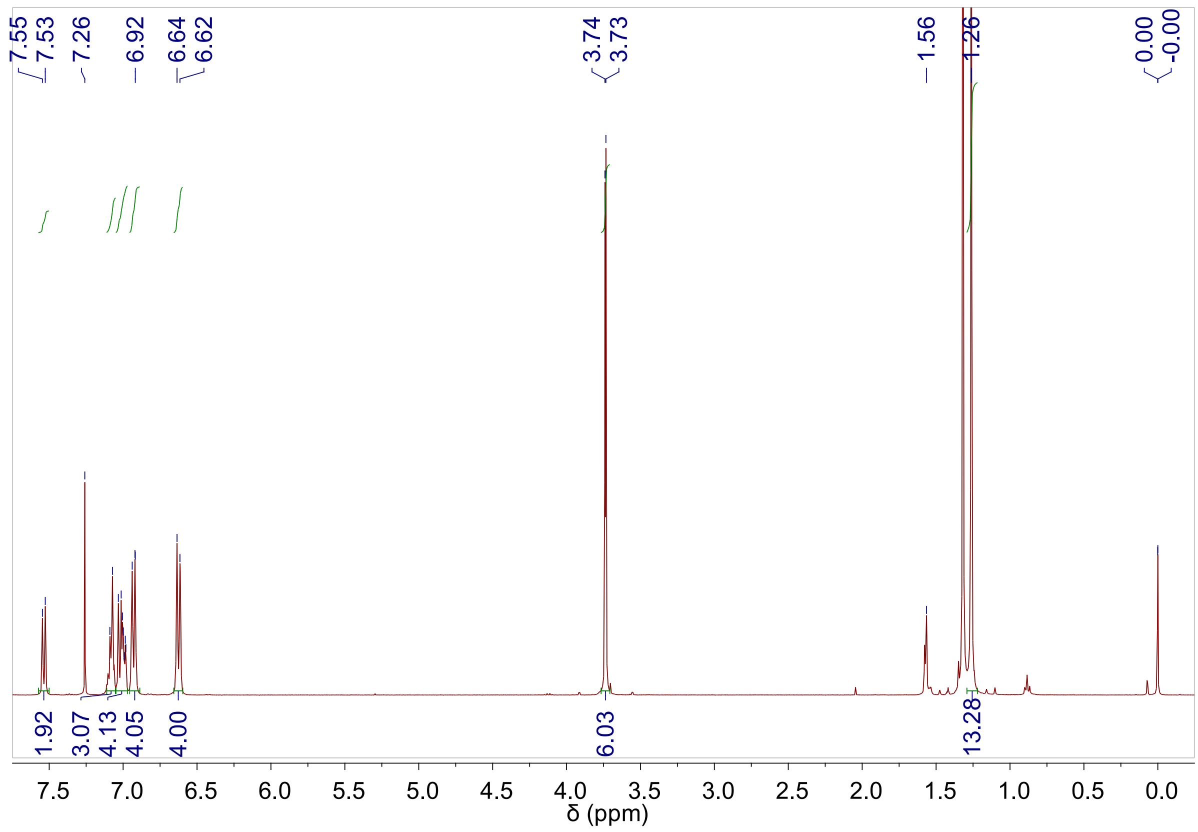

Supplementary Fig. 15. ^{1}H NMR spectrum of 2-(4-(2,2-bis(4-methoxyphenyl)-1-phenylvinyl)phenyl)-4,4,5,5-tetramethyl-1,3,2-dioxaborolane in $CDCl_3$ at 298 K.

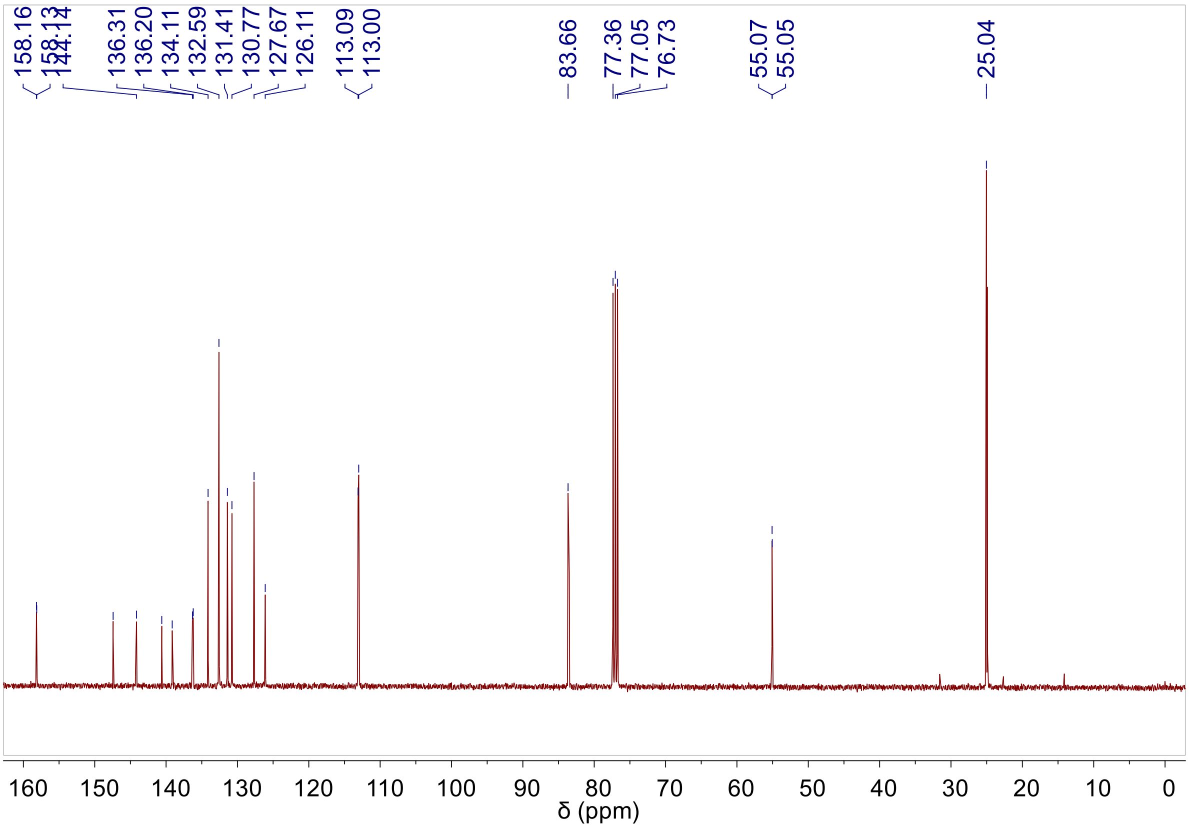

Supplementary Fig. 16. ^{13}C NMR spectrum of 2-(4-(2,2-bis(4-methoxyphenyl)-1-phenylvinyl)phenyl)-4,4,5,5-tetramethyl-1,3,2-dioxaborolane in $CDCl_3$ at 298 K.

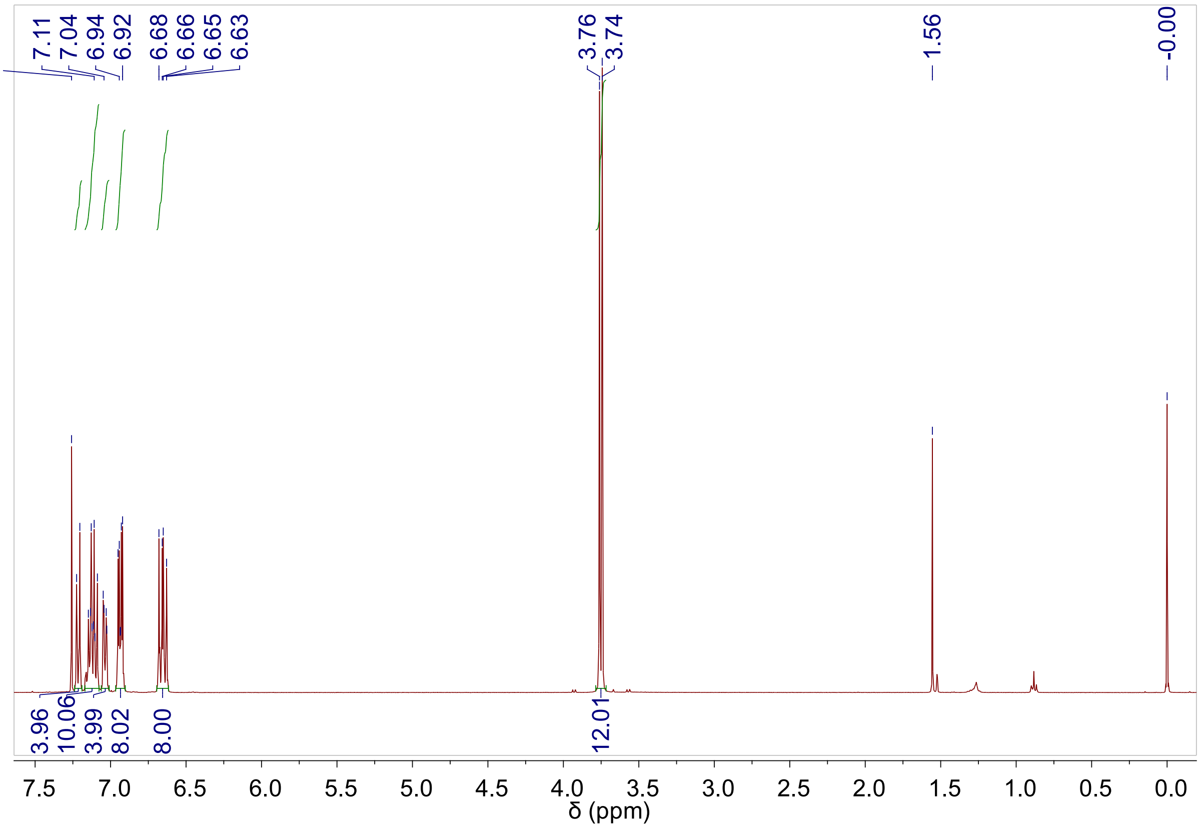

Supplementary Fig. 17. ^{1}H NMR spectrum of 2,5-bis(4-(2,2-bis(4-methoxyphenyl)-1-phenylvinyl)phenyl)-3,4-dinitrothiophene in $CDCl_3$ at 298 K.

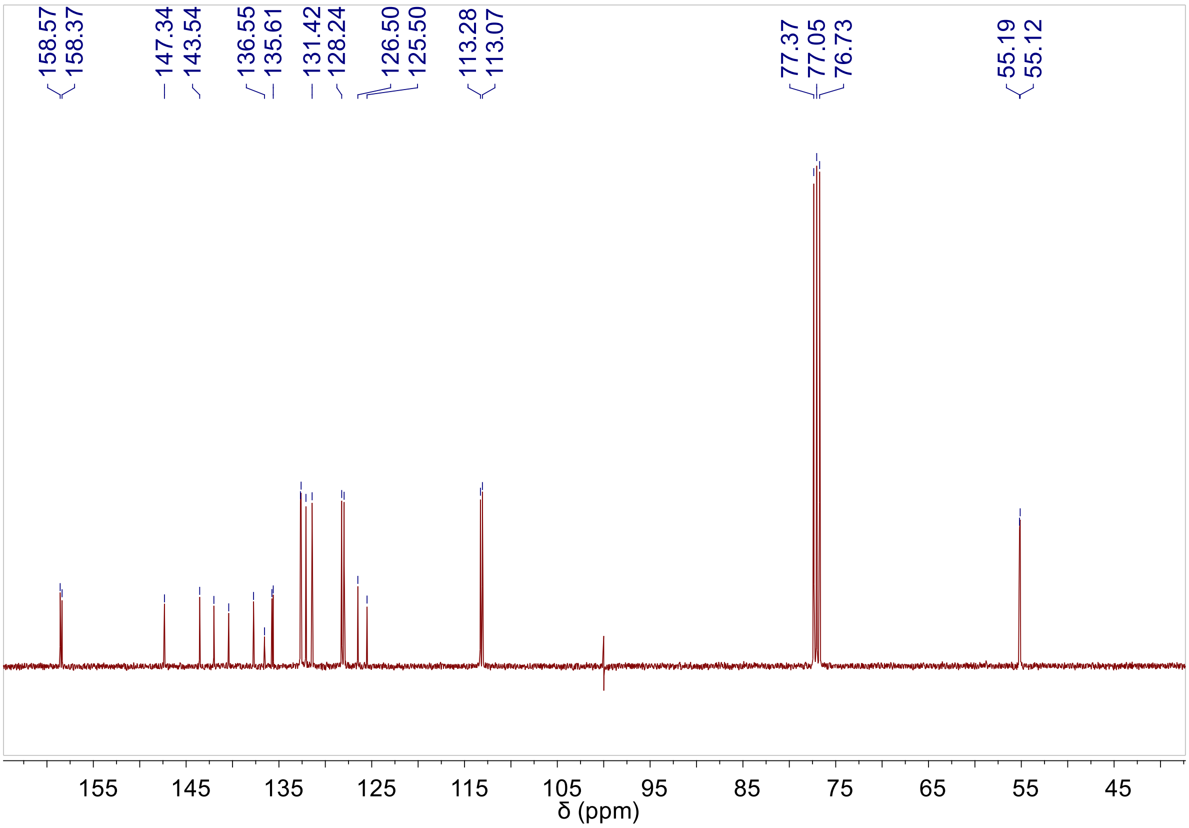

Supplementary Fig. 18. ^{13}C NMR spectrum of 2,5-bis(4-(2,2-bis(4-methoxyphenyl)-1-phenylvinyl)phenyl)-3,4-dinitrothiophene in $CDCl_3$ at 298 K.

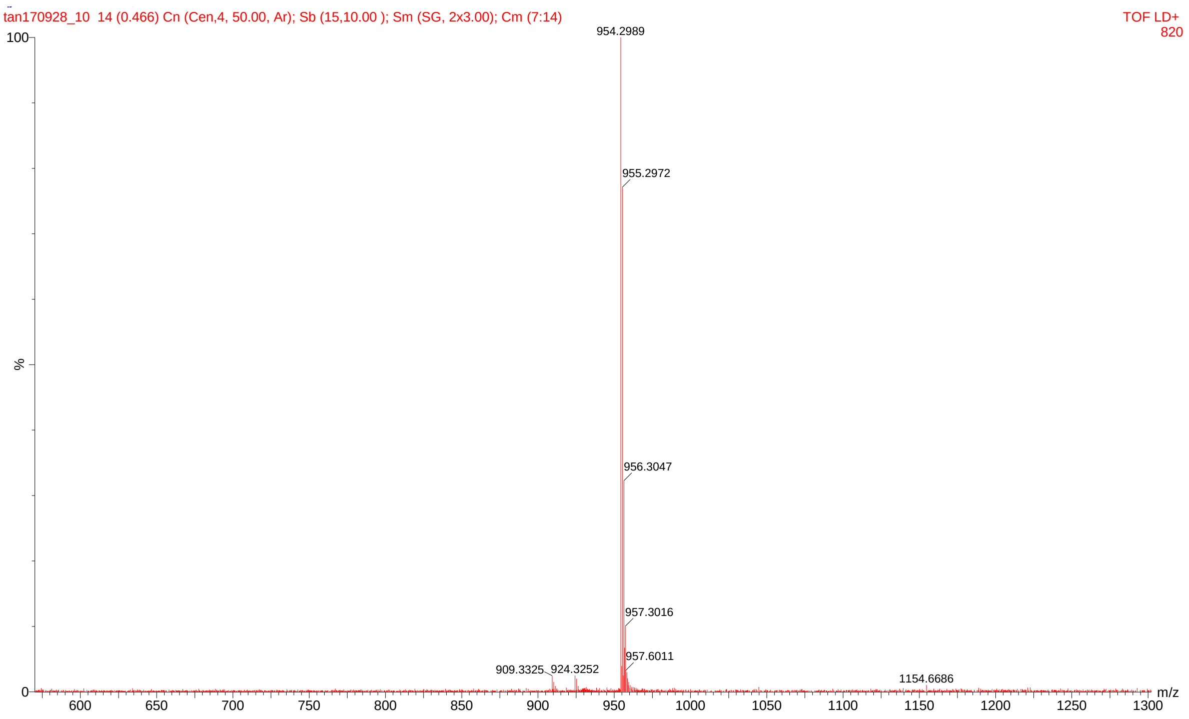

Supplementary Fig. 19. HRMS of 2,5-bis(4-(2,2-bis(4-methoxyphenyl)-1-phenylvinyl)phenyl)-3,4-dinitrothiophene.

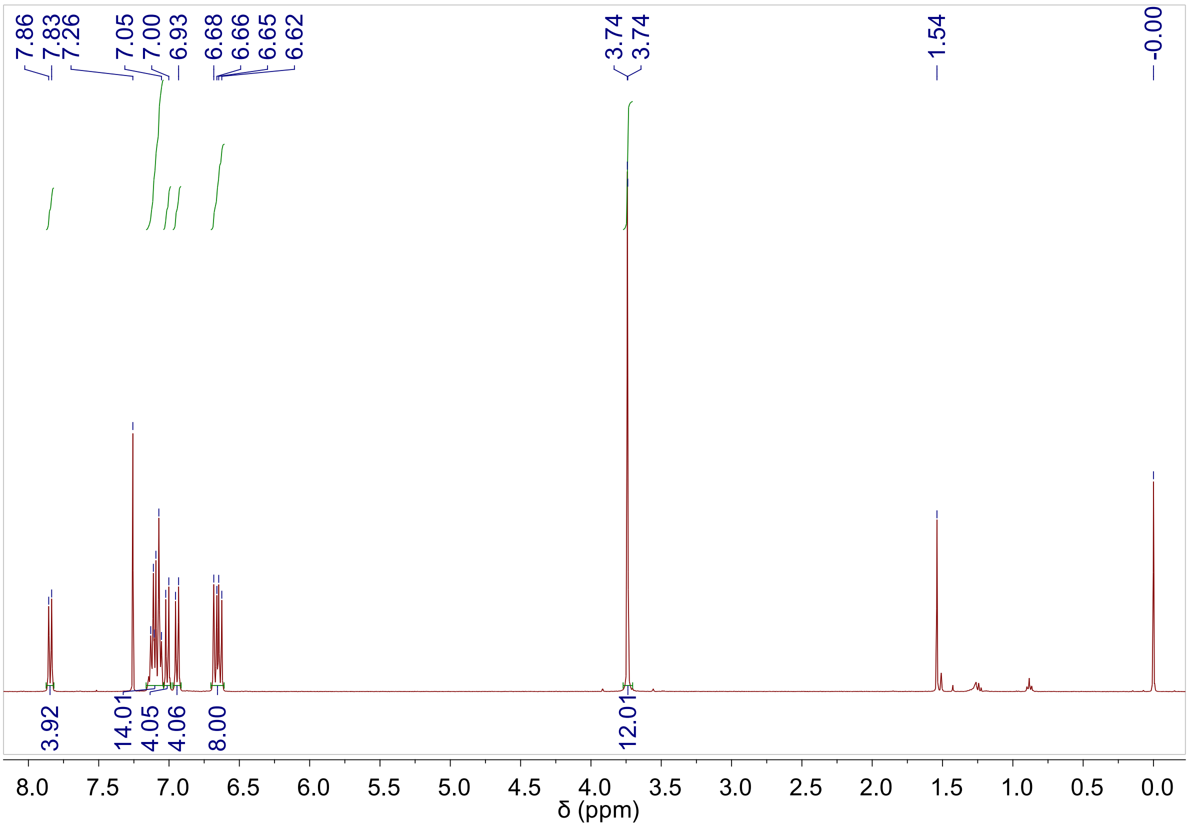

Supplementary Fig. 20. ^{1}H NMR spectrum of MTPE-TT in $CDCl_3$ at 298 K.

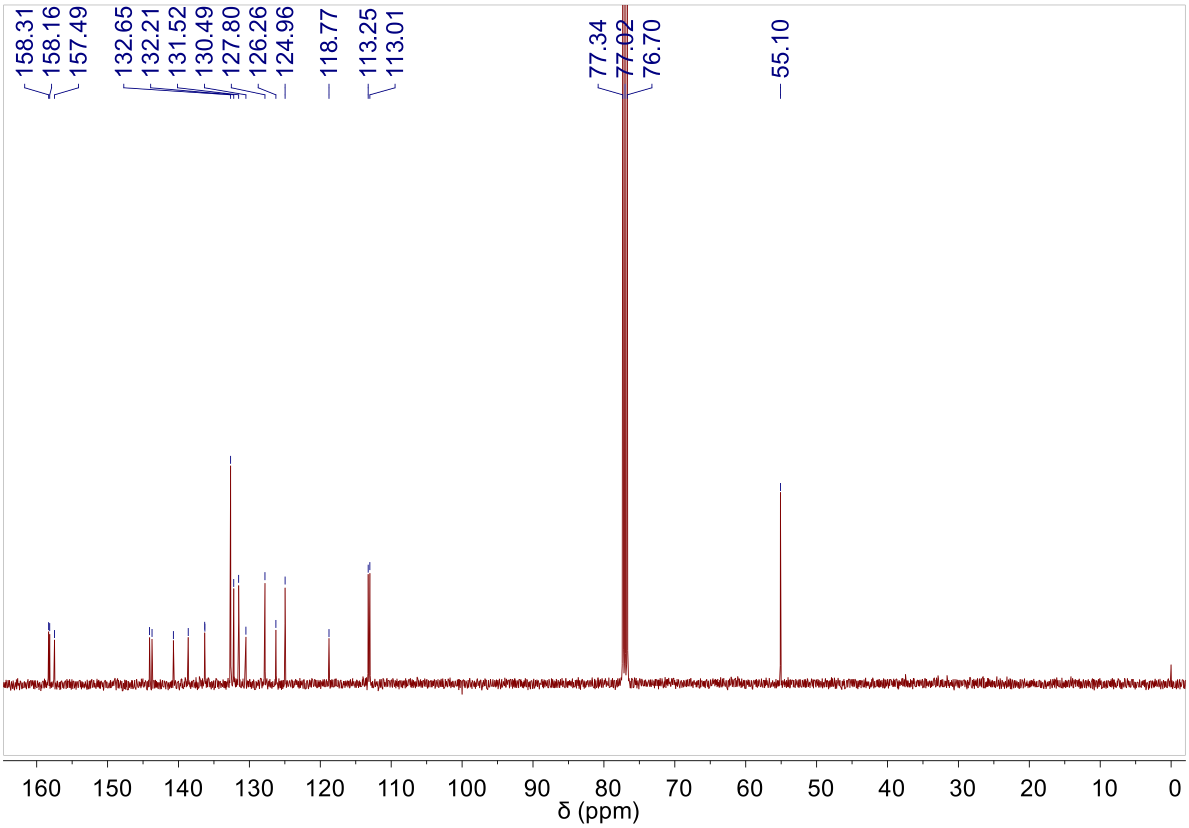

Supplementary Fig. 21. ^{13}C NMR spectrum of MTPE-TT in $CDCl_3$ at 298 K.

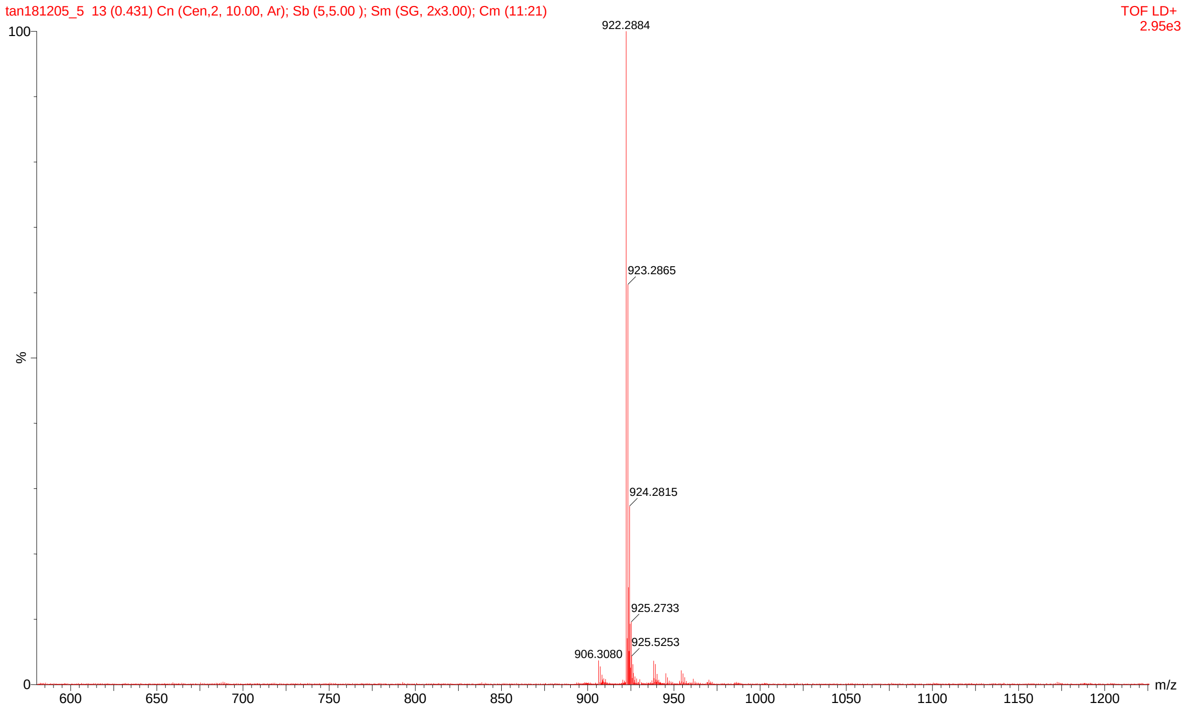

Supplementary Fig. 22. HRMS of MTPE-TT.

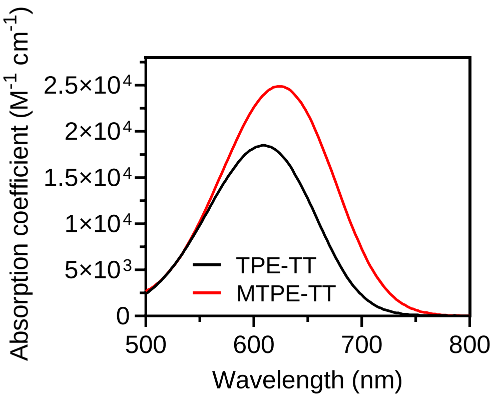

Supplementary Fig. 23. Absorption spectra of TPE-TT and MTPE-TT in THF. Experiment was repeated three times independently with similar results. Source data are provided as a Source Data file.

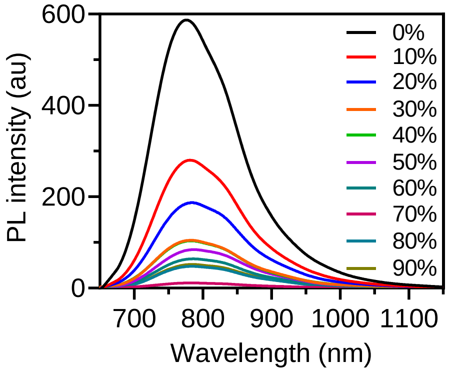

Supplementary Fig. 24. PL spectra of TPE-TT in THF/water mixtures with various water fractions (f_w) as indicated. Experiment was repeated three times independently with similar results. Source data are provided as a Source Data file.

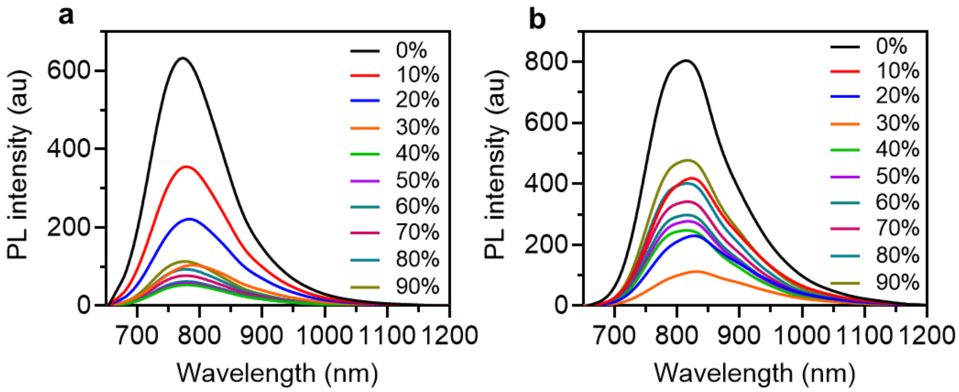

Supplementary Fig. 25. PL spectra of (a) TPE-TT and (b) MTPE-TT in DMF/glycerol mixtures with various glycerol fractions (f_g) as indicated. Experiment was repeated three times independently with similar results. Source data are provided as a Source Data file.

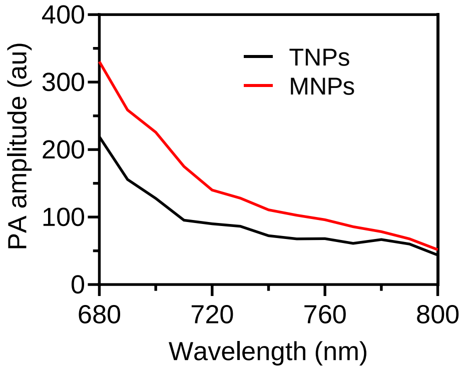

Supplementary Fig. 26. PA spectra of TNPs and MNPs in the same concentration. Experiment was repeated three times independently with similar results. Source data are provided as a Source Data file.

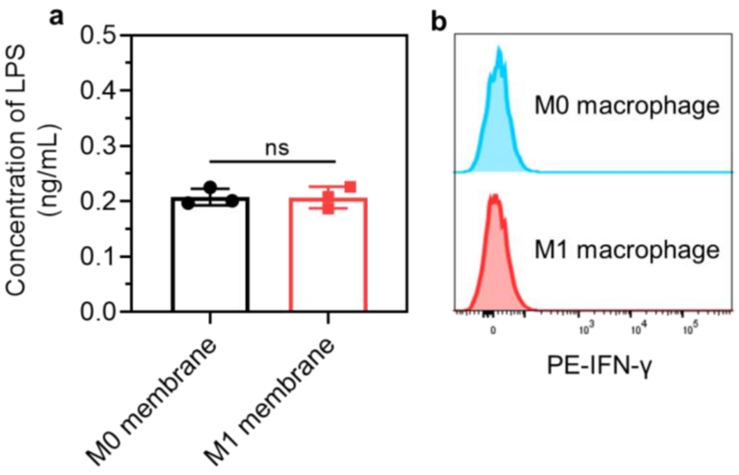

Supplementary Fig. 27. a The concentration of LPS in M0 and M1 macrophage membrane measured by LPS ELISA kit. Data are presented as mean ± SD (n = 3 independent experiments). Statistical significance was determined using two-tailed Student's t test. **b** Flow cytometry analysis of the IFN-γ level in M0 and M1 macrophages. Experiment was repeated three times independently with similar results. Source data are provided as a Source Data file.

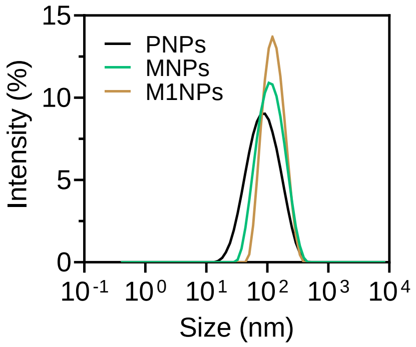

Supplementary Fig. 28. Representative DLS results of PNPs, MNPs and M1NPs. Experiment was repeated three times independently with similar results. Source data are provided as a Source Data file.

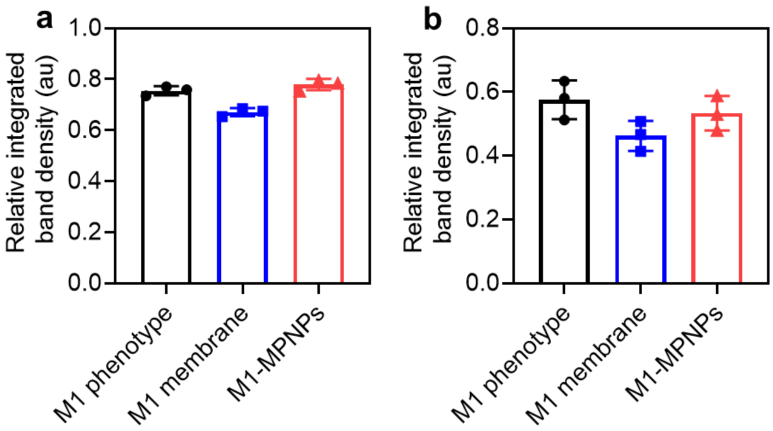

Supplementary Fig. 29. Quantification analyses of **a** CD86 and **b** iNOS expression in different formulations according to the western blots in Fig. 4g. Data are presented as mean ± SD (n = 3 independent experiments). Source data are provided as a Source Data file.

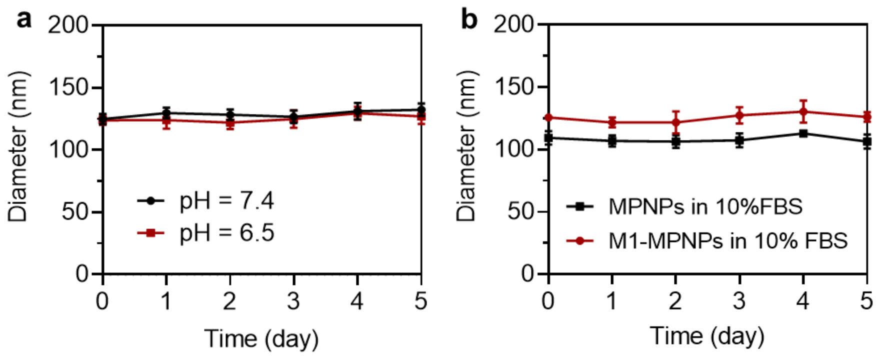

Supplementary Fig. 30. The colloidal stability of **a** M1-MPNPs in different pHs, and **b** MPNPs and M1-MPNPs in serum measured by DLS. Data are presented as mean ± SD (n = 3 independent experiments). Source data are provided as a Source Data file.

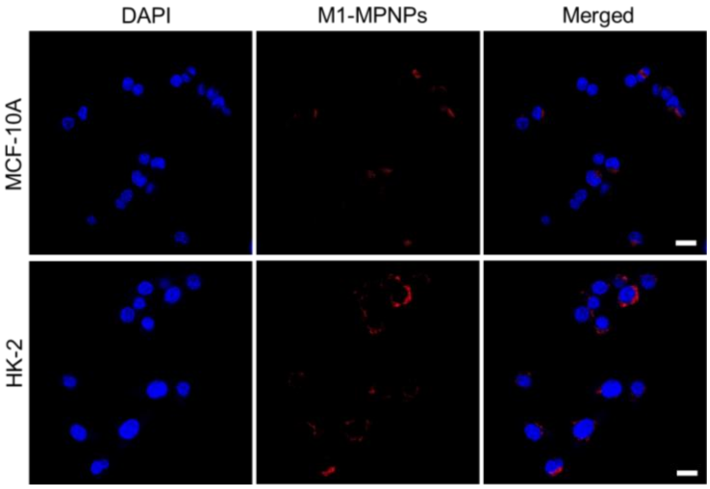

Supplementary Fig. 31. Representative CLSM images of MCF-10A and HK-2 epithelial cells after incubation with M1-MPNPs for 4 h. Scale bars: 20 µm. Experiment was repeated three times independently with similar results.

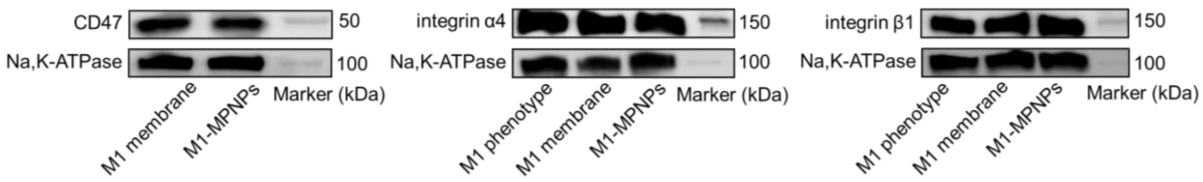

Supplementary Fig. 32. Representative western blots of CD47, α4 and β1 integrin expression in M1 macrophage membrane and M1-MPNPs. Experiment was repeated three times independently with similar results.

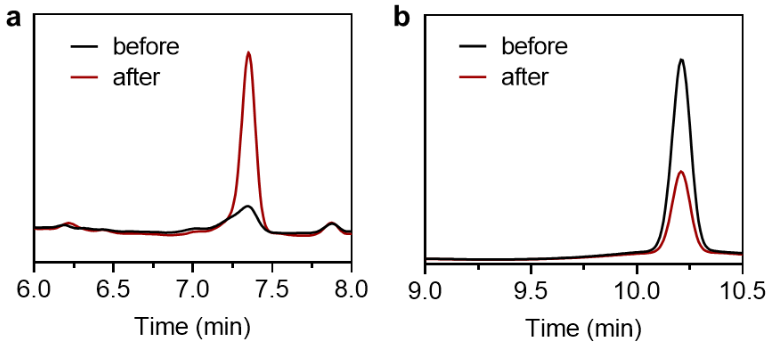

Supplementary Fig. 33. HPLC results indicating PDT effect could accelerate the conversion of PTX-NB to free PTX in cells. M1-MPNPs were incubated with 4T1 cancer cells at 37 °C for 4 h, and then the cells were washed with PBS and exposed to white light (10 mW cm^{-2}) for 5 min. The cell medium before and after light irradiation was measured by HPLC analysis. The peak of PTX-NB (b) decreased while the peak representing PTX (a) obviously increased when compared to that of cells without light irradiation. Experiment was repeated three times independently with similar results. Source data are provided as a Source Data file.

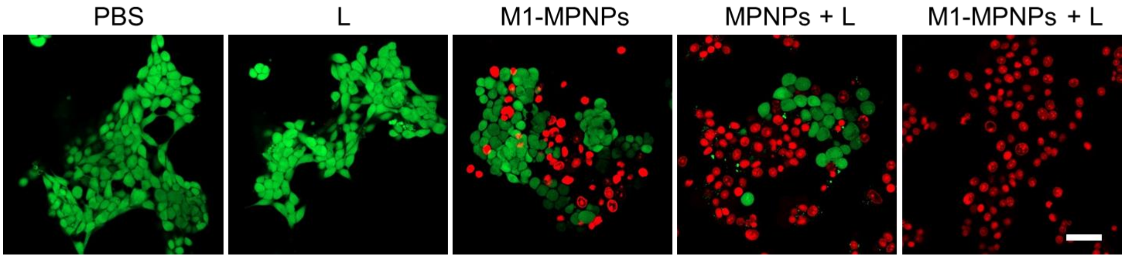

Supplementary Fig. 34. Representative live/dead staining of 4T1 cells after different treatments under light irradiation indicated by calcein-AM (green, live cells) and PI (red, dead cells) staining. Scale bars: 50 μm. Experiment was repeated three times independently with similar results.

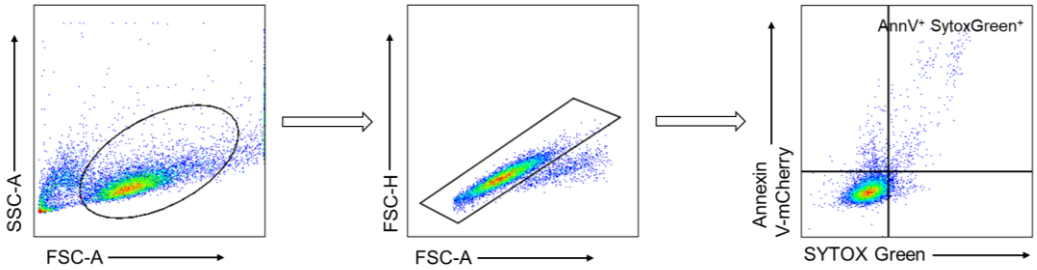

Supplementary Fig. 35. Gating scheme for the flow cytometry analysis of the population of annexin-V and Sytox Green co-staining 4T1 cells in Supplementary Fig. 36.

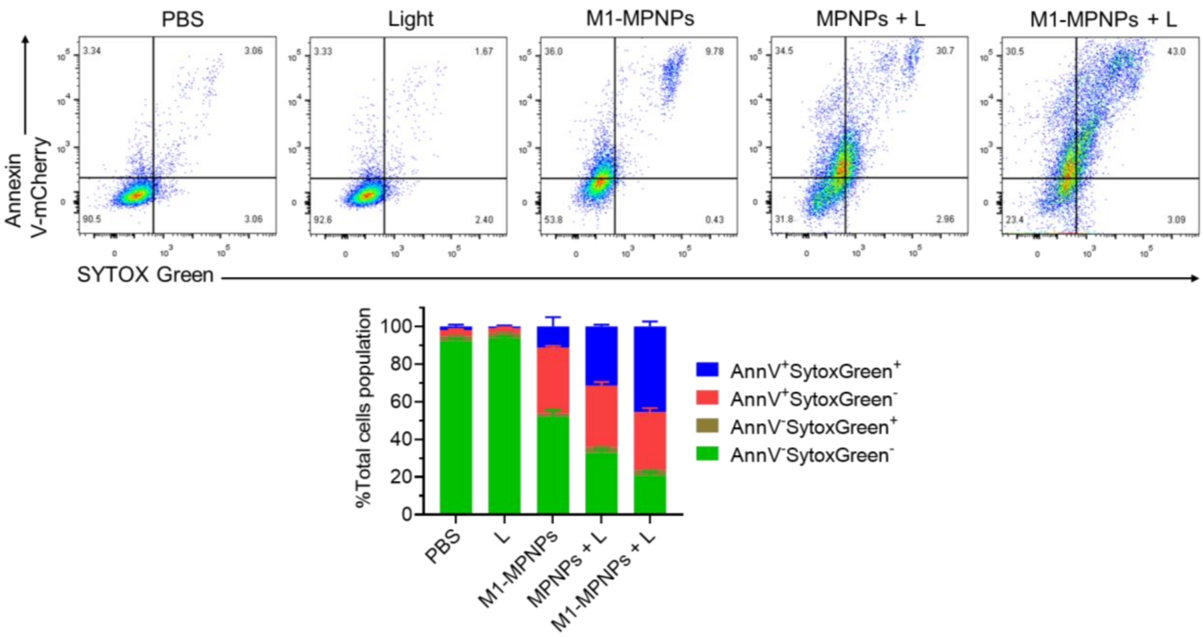

Supplementary Fig. 36. Representative flow cytometry results and quantitative analyses of the population of annexin-V and Sytox Green co-staining 4T1 cells after various treatments. Data are presented as mean ± SD (n = 3 independent experiments).

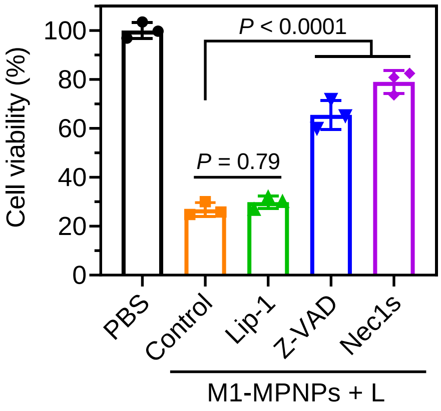

Supplementary Fig. 37. Cell viability of the 4T1 tumor cells with the treatment of “M1-MPNPs + L” after adding different kinds of cell death inhibitors. No “M1-MPNPs + L” treatment and inhibitor were applied for in the PBS group, and no inhibitor was added in the Control group. Data are presented as mean ± SD (n = 3 independent experiments). Statistical significance was determined using one-way ANOVA. Source data are provided as a Source Data file.

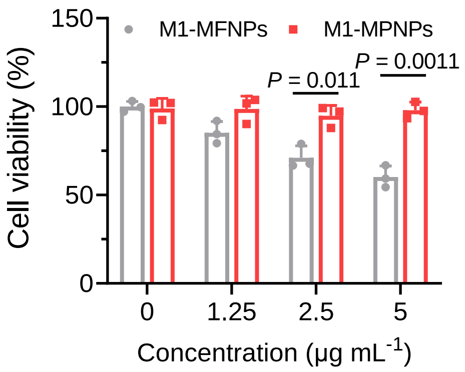

Supplementary Fig. 38. MTT assay of MCF-10A cells with the treatment of different concentraitons of M1-MFNPs or M1-MPNPs. Data are presented as mean ± SD (n = 3 independent experiments). Statistical significance was determined using two-tailed Student's t test. Source data are provided as a Source Data file.

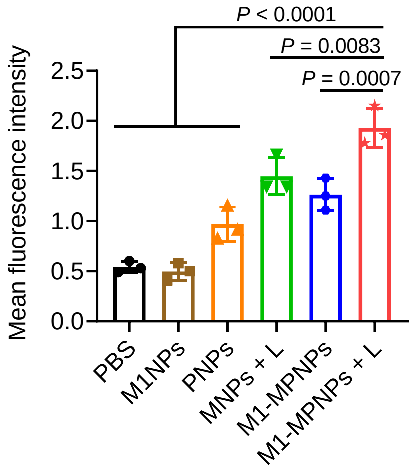

Supplementary Fig. 39. Quantitative analyses of the proportions of CRT exposure in 4T1 tumor cells in various groups according to the immunostaining results in Fig. 5f. As compared with the PNPs-treated cells, the M1-MPNPs-treated cells exhibited higher CRT exposure, which was probably due to the enhanced tumor-targeting ability mediated by M1 macrophage membrane coating. Data are presented as mean ± SD (n = 3 independent experiments). Statistical significance was determined using one-way ANOVA. Source data are provided as a Source Data file.

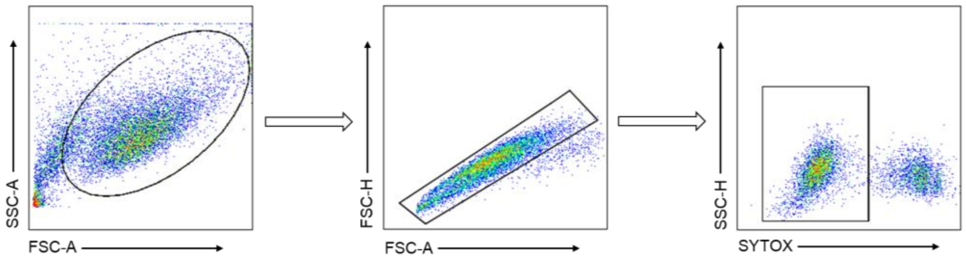

Supplementary Fig. 40. Gating scheme for the flow cytometry analysis of the proportions of calreticulin exposure in Fig. 5g.

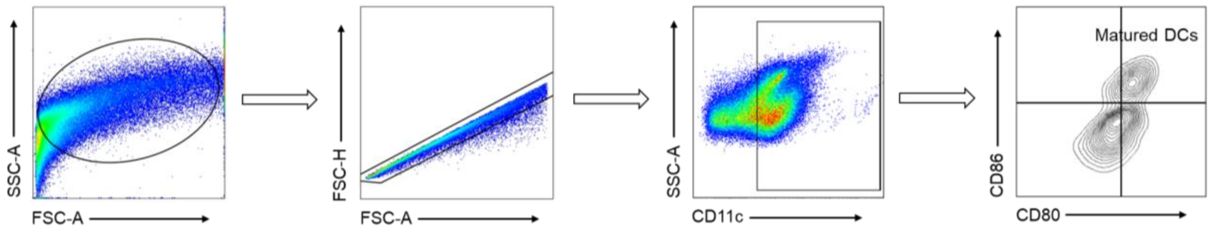

Supplementary Fig. 41. Gating scheme for the flow cytometry analysis of the population of BMDCs maturation (CD11c$^+$CD80$^+$CD86$^+$) in Supplementary Fig. 42.

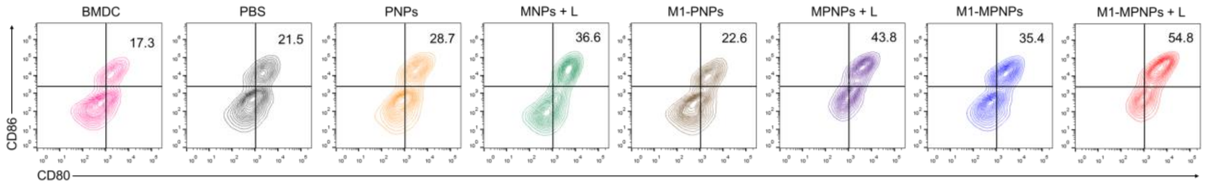

Supplementary Fig. 42. Representative flow cytometry analysis of the population of BMDCs maturation (CD11c$^+$CD80$^+$CD86$^+$) after various treatments. Experiment was repeated three times independently with similar results.

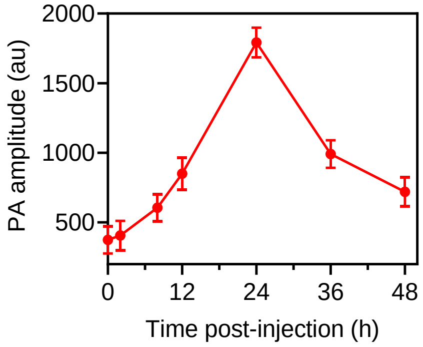

Supplementary Fig. 43. PA amplitude of tumor site under 680 nm excitation at different time points after i.v injection of M1-MPNPs into tumor-bearing mice. Data are presented as mean ± SD (n = 3 mice). Source data are provided as a Source Data file.

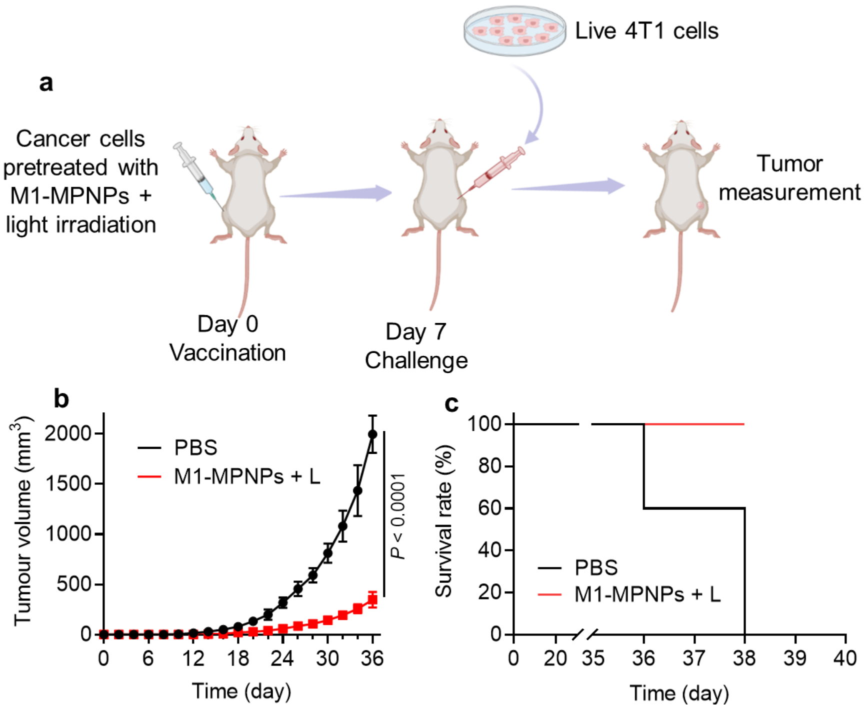

Supplementary Fig. 44. a Schematic illustration of in vivo prophylactic vaccination model experiment schedule. The illustration was created with BioRender.com. **b** The volumes of the right tumors from the mice immunized with PBS or M1-MPNPs + light-treated cancer cells after the challenge with live cancer cells. Data are presented as mean ± SD (n = 5 mice). Statistical significance was determined using two-tailed Student's t test. **c** Survival curves of the mice immunized with PBS or M1-MPNPs + light-treated cancer cells after the challenge with live cancer cells (n = 5 mice). Source data are provided as a Source Data file.

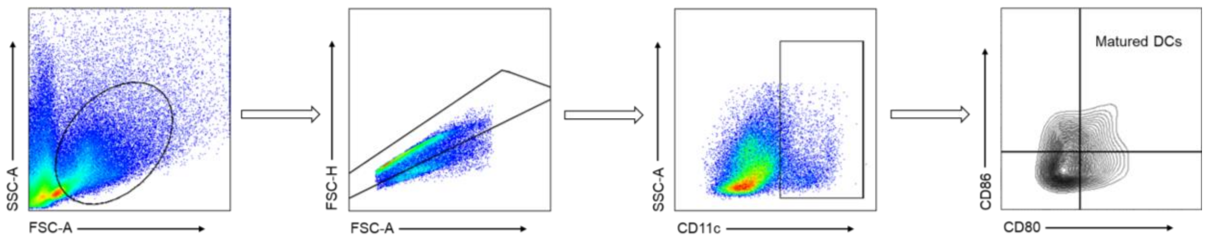

Supplementary Fig. 45. Gating scheme for the flow cytometry analysis of the proportions of DC maturation in lymph nodes of 4T1 tumor-bearing mice in Supplementary Fig. 46.

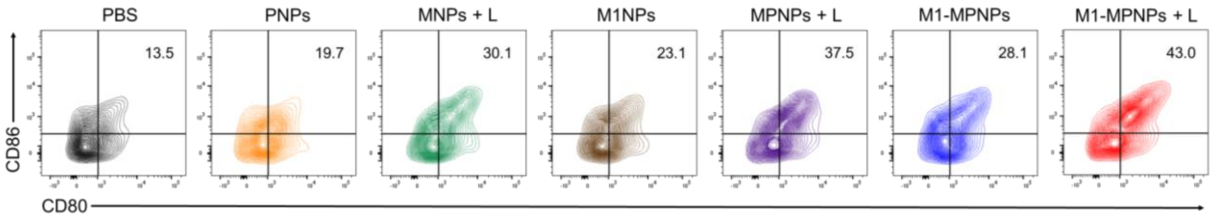

Supplementary Fig. 46. Representative flow cytometry plots displaying the proportions of DC maturation in lymph nodes of 4T1 tumor-bearing mice in various groups on day 14. Experiment was repeated three times independently with similar results.

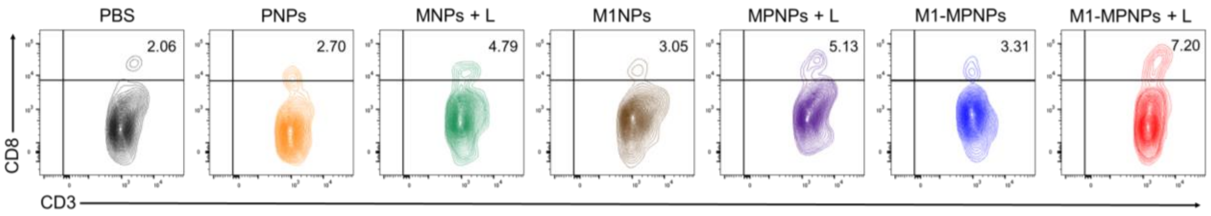

Supplementary Fig. 47. Representative flow cytometry plots displaying the proportions of tumor-infiltrating $CD8^+$ T cells of 4T1 tumor-bearing mice in various groups on day 14. Experiment was repeated three times independently with similar results.

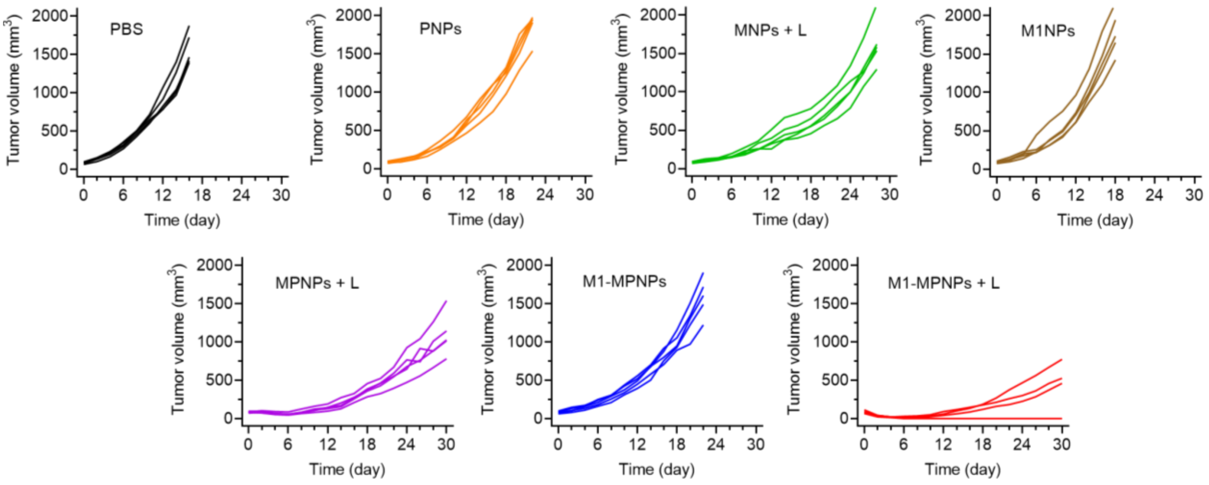

Supplementary Fig. 48. Individual tumor volume of the primary tumors of the bilateral 4T1 tumor-bearing mice in various groups. The tumor growth curves were monitored for 30 days (*n* = 5 mice). Source data are provided as a Source Data file.

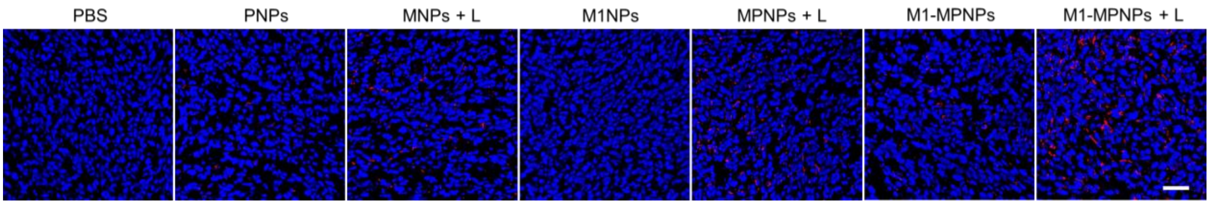

Supplementary Fig. 49. Representative CLSM images showing the ecto-CRT expression (red pseudocolor) in the sections of primary tumors collected from bilateral 4T1 tumor-bearing mice after various treatments (*n* = 3 mice). Scale bars: 50 µm. Experiment was repeated three times independently with similar results.

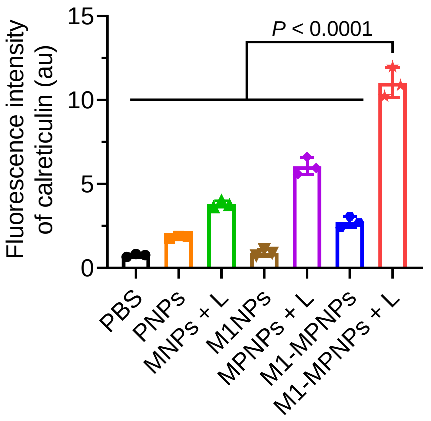

Supplementary Fig. 50. Quantitative analyses of the ecto-CRT expression in primary tumors collected from bilateral 4T1 tumor-bearing mice after various treatments. Data are presented as mean ± SD (n = 3 independent experiments). Statistical significance was determined using one-way ANOVA. Source data are provided as a Source Data file.

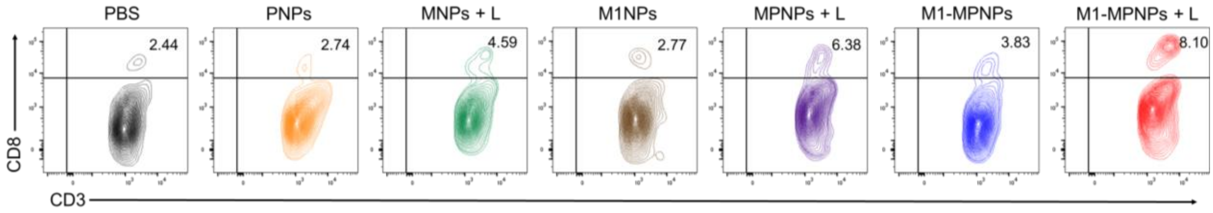

Supplementary Fig. 51. Representative flow cytometry plots displaying the proportions of tumor-infiltrating $CD8^+$ T cells in primary tumors collected from bilateral 4T1 tumor-bearing mice after various treatments. Experiment was repeated three times independently with similar results.

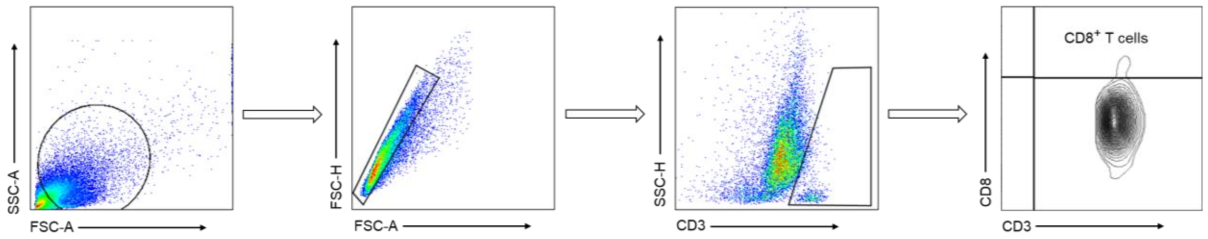

Supplementary Fig. 52. Gating scheme for the flow cytometry analysis of the proportions of $CD8^+$ T cells in the distant tumors collected from bilateral 4T1 tumor-bearing mice in Fig. 8i.

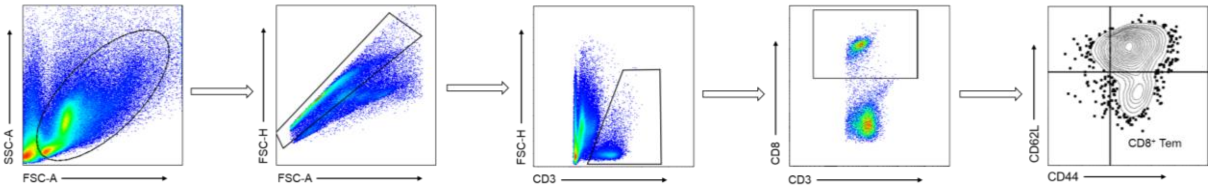

Supplementary Fig. 53. Gating scheme for the flow cytometry analysis of CD8$^+$ Tem cells (CD44$^+$ and CD62L$^-$) in the spleen of distant tumors in bilateral 4T1 tumor-bearing mice in Fig. 8k,l.

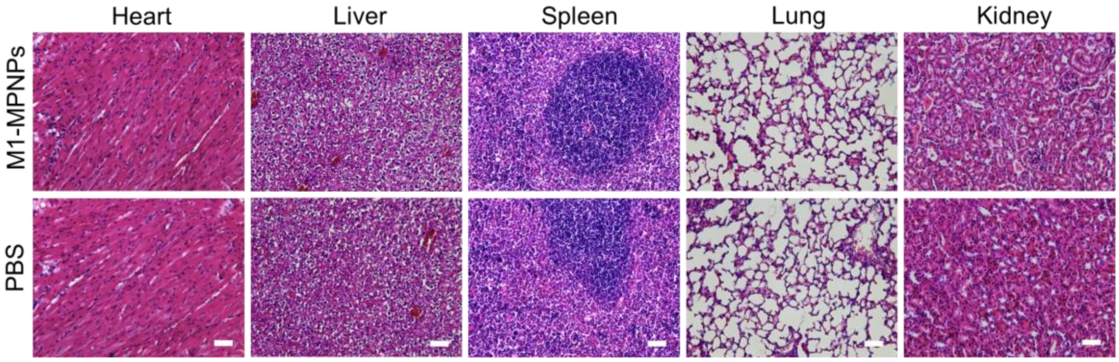

Supplementary Fig. 54. Representative H&E staining of heart, liver, spleen, lung and kidney of healthy mice with the treatment of PBS or M1-MPNPs (n = 3 mice). Scale bars: 50 μm. Experiment was repeated three times independently with similar results.

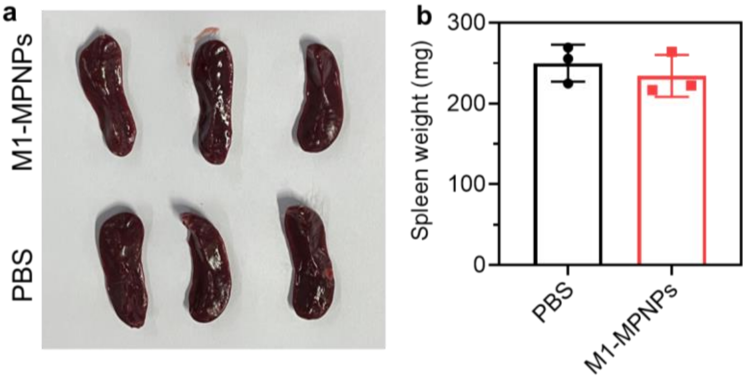

Supplementary Fig. 55. a The photograph and **b** weight of the spleens from the mice with the treatment of PBS or M1-MPNPs. Data are presented as mean ± SD (n = 3 mice). Source data are provided as a Source Data file.

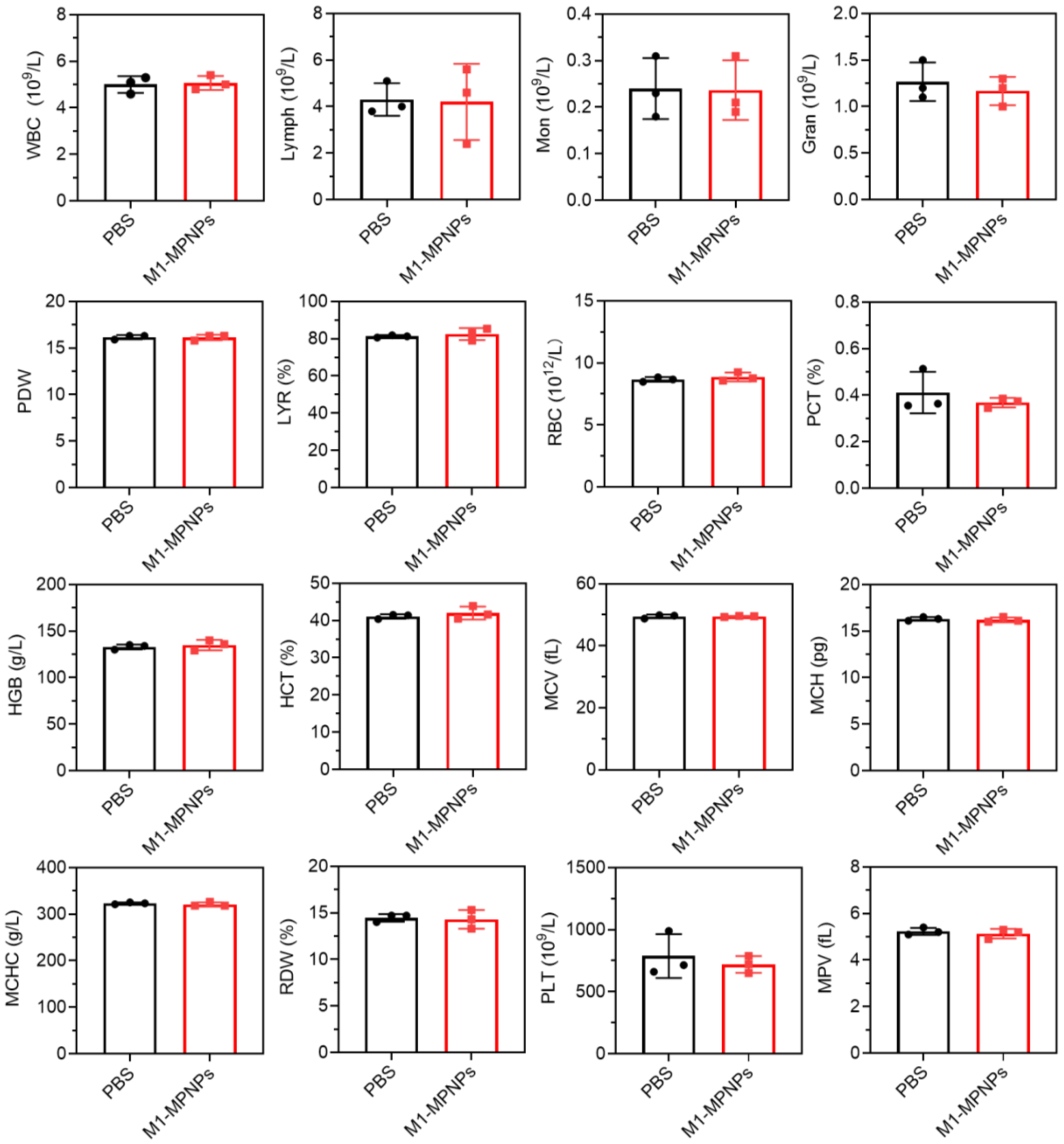

Supplementary Fig. 56. Blood routine indexes (white blood cell count (WBC), lymphocyte (Lymph), monocytes (Mon), granulocyte (Gran), platelet distribution width (PDW), Lymphocyte ratio (LYR), red blood cell count (RBC), plateletcrit (PCT), hemoglobin (HGB), hematocrit (HCT), mean corpuscular volume (MCV), mean corpuscular hemoglobin (MCH), mean corpuscular concentration (MCHC), red blood cell distribution width (RDW), platelets (PLT), and mean platelet volume (MPV)) of the healthy mice with the treatment of PBS or M1-MPNPs. Data are presented as mean ± SD (n = 3 mice). Source data are provided as a Source Data file.

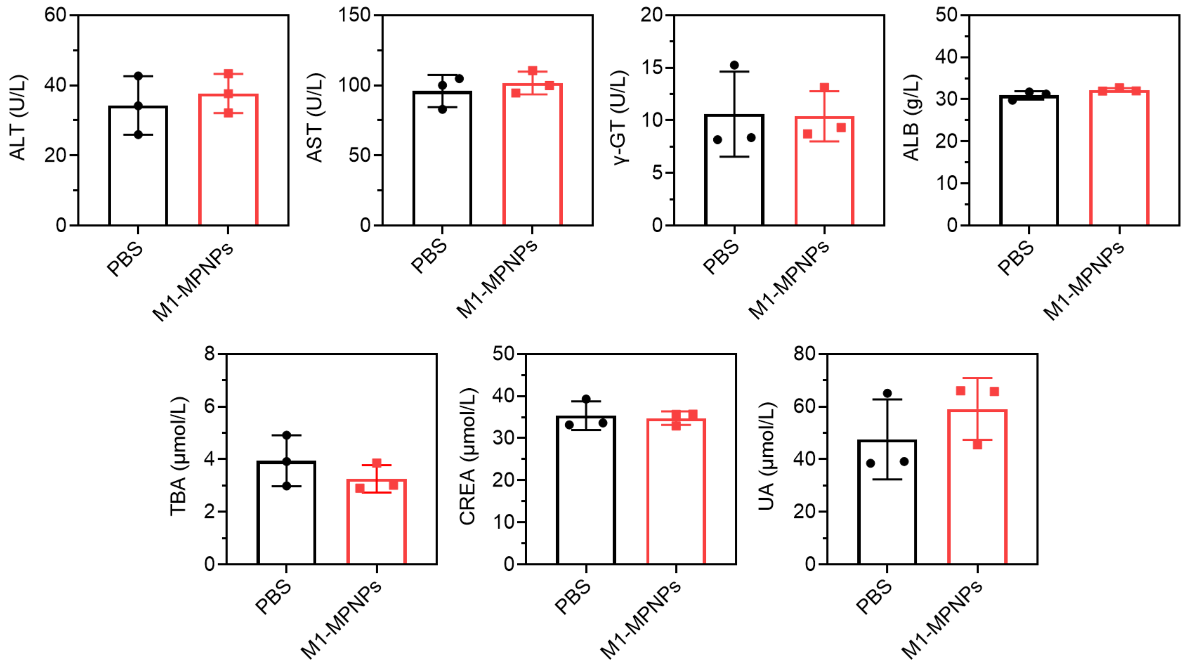

Supplementary Fig. 57. Blood test parameters included liver and renal function indexes (alanine transaminase (ALT), aspartate transaminase (AST), γ-glutamyl transpeptadase (γ-GT), albumin (ALB), total bile acid (TBA), creatinine (CREA), and uric acid (UA)) of the healthy mice with the treatment of PBS or M1-MPNPs. Data are presented as mean ± SD (n = 3 mice). Source data are provided as a Source Data file.

Supplementary Table 1. Cartesian coordinates of optimized TPE-TT calculated by the DFT, B3LYP/6-31G(d), Gaussian 09 program.

Atom	x	y	z
C	-3.66	2.649	-0.741
C	-4.995	2.646	-0.702
C	-5.487	1.418	-0.484
S	-4.312	0.531	-0.375
C	-3.15	1.423	-0.551
C	-1.856	1.033	-0.515
C	-6.776	1.02	-0.382
C	-0.835	1.894	-0.689
C	0.453	1.514	-0.646
C	0.83	0.238	-0.432
C	-0.191	-0.624	-0.254
C	-1.478	-0.244	-0.295
C	-7.134	-0.264	-0.171
C	-8.416	-0.653	-0.067
C	-9.449	0.207	-0.171
C	-9.091	1.489	-0.375
C	-7.809	1.878	-0.478
C	-10.742	-0.198	-0.047
C	2.138	-0.129	-0.371
C	3.069	0.791	0.009
C	2.539	-1.393	-0.678
C	-11.022	-1.31	0.69
C	-11.762	0.482	-0.638
C	-11.97	-2.179	0.245
C	-10.347	-1.525	1.852
C	4.271	0.822	-0.628
C	2.773	1.657	1.016
C	-13.014	0.456	-0.131
C	-14.037	1.109	-0.703
C	-13.843	1.816	-1.824
C	-12.615	1.853	-2.36
C	-11.602	1.196	-1.773
C	-12.703	-2.95	1.078
C	-13.647	-3.795	0.635
C	-13.897	-3.898	-0.677
C	-13.194	-3.142	-1.531
C	-12.254	-2.3	-1.07
C	-10.119	-2.761	2.346
C	-9.438	-2.969	3.484
C	-8.952	-1.931	4.177
C	-9.153	-0.691	3.712
C	-9.835	-0.501	2.571
C	3.658	-1.928	-0.141
C	4.078	-3.168	-0.437
C	3.388	-3.925	-1.301

C	2.281	-3.42	-1.86
C	1.872	-2.179	-1.551
C	5.403	1.251	-0.029
C	6.59	1.266	-0.658
C	6.686	0.844	-1.925
C	5.584	0.404	-2.546
C	4.406	0.394	-1.903
C	3.318	2.891	1.101
C	3.009	3.748	2.087
C	2.131	3.397	3.036
C	1.567	2.184	2.977
C	1.885	1.339	1.983
N	-3.227	3.817	-0.944
S	-4.343	4.762	-1.069
N	-5.445	3.811	-0.882
H	-1.006	2.962	-0.887
H	1.2	2.303	-0.837
H	-0.004	-1.682	-0.002
H	-2.216	-1.043	-0.112
H	-6.385	-1.069	-0.083
H	-8.587	-1.734	0.069
H	-9.846	2.293	-0.405
H	-7.652	2.956	-0.623
H	-13.245	-0.074	0.809
H	-15.041	1.074	-0.247
H	-14.68	2.351	-2.302
H	-12.446	2.411	-3.296
H	-10.637	1.224	-2.307
H	-12.598	-2.877	2.174
H	-14.236	-4.393	1.35
H	-14.673	-4.588	-1.048
H	-13.384	-3.221	-2.614
H	-11.695	-1.738	-1.836
H	-10.434	-3.667	1.801
H	-9.258	-3.996	3.843
H	-8.389	-2.094	5.111
H	-8.763	0.174	4.275
H	-9.989	0.549	2.27
H	4.259	-1.381	0.605
H	4.989	-3.572	0.036
H	3.73	-4.942	-1.552
H	1.716	-4.023	-2.592
H	0.987	-1.813	-2.098
H	5.416	1.556	1.03
H	7.494	1.607	-0.126
H	7.658	0.85	-2.446
H	5.647	0.058	-3.592
H	3.538	0.05	-2.491
H	4.001	3.275	0.325
H	3.463	4.753	2.109

H	1.871	4.098	3.846
H	0.846	1.879	3.754
H	1.405	0.347	2.022

Supplementary Table 2. Cartesian coordinates of optimized MTPE-TT calculated by the DFT, B3LYP/6-31G(d), Gaussian 09 program.

Atom	x	y	z
H	5.664	4.54	1.657
H	6.201	2.977	-0.027
H	5.708	-0.363	-2.719
H	7.797	-0.495	-3.741
H	9.623	1.154	-0.314
H	7.531	1.243	0.774
H	3.041	-2.084	-2.328
H	3.688	-4.34	-2.719
H	5.64	-5.298	-1.598
H	6.919	-3.918	-0.034
H	6.268	-1.678	0.432
H	-7.782	0.917	1.904
H	-6.54	0.584	3.847
H	-7.267	-3.567	3.649
H	-8.476	-3.273	1.645
H	-9.742	-1.447	-2.053
H	-11.493	-2.831	-2.721
H	-12.312	-3.804	1.302
H	-10.587	-2.361	2.016
H	-8.511	1.423	-2.692
H	-10.276	2.651	-3.711
H	-12.491	2.754	-2.681
H	-12.879	1.6	-0.559
H	-11.124	0.415	0.525
H	-5.423	3.099	-1.116
H	-7.643	2.547	-0.85
H	-6.562	-1.51	-0.212
H	-4.334	-0.953	-0.411
H	-0.168	-1.109	-0.427
H	2.013	-1.832	-0.255
H	3.393	2.062	-1.238
H	1.217	2.807	-1.351
C	10.332	-0.277	-3.929
O	10.158	0.298	-2.655
C	2.955	4.09	4.5
O	3.976	4.309	3.554
C	-5.396	-0.753	5.695
O	-6.101	-1.802	5.071
C	-13.472	-4.413	-2.427
O	-13.088	-4.293	-1.076
N	-3.182	3.843	-1.421
S	-2.039	4.737	-1.642
N	-0.966	3.75	-1.473
C	3.962	1.212	1.636
C	3.651	2.119	2.576

C	4.245	3.325	2.634
C	5.163	3.556	1.678
C	5.478	2.651	0.739
C	6.569	-0.021	-2.119
C	7.768	-0.085	-2.718
C	8.895	0.338	-2.116
C	8.73	0.818	-0.87
C	7.532	0.886	-0.27
C	3.898	-2.464	-1.747
C	4.261	-3.732	-1.998
C	5.335	-4.258	-1.394
C	6.036	-3.497	-0.544
C	5.661	-2.23	-0.305
C	-7.682	-0.127	2.248
C	-6.978	-0.311	3.377
C	-6.815	-1.528	3.93
C	-7.394	-2.541	3.261
C	-8.099	-2.359	2.134
C	-10.307	-1.945	-1.246
C	-11.305	-2.754	-1.638
C	-12.047	-3.455	-0.759
C	-11.72	-3.277	0.533
C	-10.722	-2.471	0.927
C	-9.467	1.471	-2.143
C	-10.456	2.149	-2.745
C	-11.674	2.201	-2.19
C	-11.883	1.562	-1.032
C	-10.884	0.886	-0.443
C	4.891	1.438	0.683
C	6.4	0.473	-0.875
C	-8.276	-1.141	1.585
C	-9.97	-1.776	0.049
C	-9.641	0.822	-0.971
C	-8.967	-0.94	0.43
C	4.578	-1.672	-0.89
C	5.18	0.517	-0.275
C	4.222	-0.382	-0.644
C	-8.646	0.121	-0.364
C	-5.628	2.036	-0.924
C	-6.924	1.712	-0.792
C	-7.338	0.457	-0.526
C	-6.343	-0.444	-0.398
C	-5.047	-0.119	-0.529
C	0.605	-0.348	-0.633
C	1.874	-0.779	-0.555
C	2.931	0.033	-0.75
C	2.611	1.313	-1.027
C	1.341	1.743	-1.107
C	-4.633	1.137	-0.796
C	0.283	0.933	-0.91

C	-0.994	1.375	-0.976
S	-2.194	0.541	-0.772
C	-3.328	1.473	-0.919
C	-2.783	2.668	-1.19
C	-1.449	2.611	-1.223
H	2.885	1.832	3.315
H	3.439	0.244	1.704
H	-14.338	-5.112	-2.473
H	-13.799	-3.427	-2.825
H	-12.643	-4.849	-3.027
H	-4.857	-1.18	6.571
H	-4.643	-0.325	4.997
H	-6.104	0.021	6.068
H	2.875	5.003	5.132
H	1.98	3.934	3.987
H	3.218	3.233	5.161
H	11.416	-0.236	-4.176
H	10.017	-1.345	-3.917
H	9.779	0.309	-4.697
