# Supplementary material for: A photo-triggered self-accelerated nanoplatform for multifunctional image-guided combination cancer immunotherapy
Source: Nat Commun. 2023 Aug 25;14:5216. doi: 10.1038/s41467-023-40996-2 (PMC10457322; doi:10.1038/s41467-023-40996-2)
Supplement: Supplementary file 3 — Reporting Summary [file 41467_2023_40996_MOESM3_ESM.pdf]

nature portfolio

Corresponding author(s): Wen Li, Ji Qi, Ben Zhong Tang

Last updated by author(s): Aug 2, 2023

Reporting Summary

Nature Portfolio wishes to improve the reproducibility of the work that we publish. This form provides structure for consistency and transparency in reporting. For further information on Nature Portfolio policies, see our Editorial Policies and the Editorial Policy Checklist.

Statistics

For all statistical analyses, confirm that the following items are present in the figure legend, table legend, main text, or Methods section.

n/a	Confirmed	
☐	☒	The exact sample size (*n*) for each experimental group/condition, given as a discrete number and unit of measurement
☐	☒	A statement on whether measurements were taken from distinct samples or whether the same sample was measured repeatedly
☐	☒	The statistical test(s) used AND whether they are one- or two-sided<br>*Only common tests should be described solely by name; describe more complex techniques in the Methods section.*
☒	☐	A description of all covariates tested
☐	☒	A description of any assumptions or corrections, such as tests of normality and adjustment for multiple comparisons
☐	☒	A full description of the statistical parameters including central tendency (e.g. means) or other basic estimates (e.g. regression coefficient) AND variation (e.g. standard deviation) or associated estimates of uncertainty (e.g. confidence intervals)
☐	☒	For null hypothesis testing, the test statistic (e.g. *F*, *t*, *r*) with confidence intervals, effect sizes, degrees of freedom and *P* value noted<br>*Give P values as exact values whenever suitable.*
☒	☐	For Bayesian analysis, information on the choice of priors and Markov chain Monte Carlo settings
☒	☐	For hierarchical and complex designs, identification of the appropriate level for tests and full reporting of outcomes
☒	☐	Estimates of effect sizes (e.g. Cohen's *d*, Pearson's *r*), indicating how they were calculated

Our web collection on statistics for biologists contains articles on many of the points above.

Software and code

Policy information about availability of computer code

Data collection	TEM: 120KV Transmission electron microscope (HT7800); Flow Cytometry: FACSAria III; Confocal Imaging: LSM 800 with Airyscan; In-vivo Imaging: NightOWL Ⅱ LB983; DLS: Nano-ZS; 1H, 13C NMR spectra: Bruker-DPX 400 spectrometer; HPLC: Shimadzu LC-20A; UV-vis: Shimadzu UV-1800 spectrometer; FL: HITACHI F-7000 spectrofluorometer; HRMS: Varian 7.0T FTMS Mass Spectrometer System; DFT: Gaussian 09 program package (revision D. 01) at the level of B3LYP/6-31G*; PA: small-animal opt-acoustic tomography system (MOST, iTheraMedical).
Data analysis	Graphpad Prism (version 8.0); Image J2x (version 2.1.4.7); FlowJo (version 10.0); PA image: View MSOT software suite (V3.6, iThera Medical); FL image: Indigo; MestReNova (version 14.0); GaussView (version 5.0).

For manuscripts utilizing custom algorithms or software that are central to the research but not yet described in published literature, software must be made available to editors and reviewers. We strongly encourage code deposition in a community repository (e.g. GitHub). See the Nature Portfolio guidelines for submitting code & software for further information.

Data

Policy information about availability of data

All manuscripts must include a data availability statement. This statement should provide the following information, where applicable:

- Accession codes, unique identifiers, or web links for publicly available datasets
- A description of any restrictions on data availability
- For clinical datasets or third party data, please ensure that the statement adheres to our policy

All data are available within the Article, Supplementary Information or Source Data file.

Human research participants

Policy information about studies involving human research participants and Sex and Gender in Research.

Reporting on sex and gender	not applicable
Population characteristics	not applicable
Recruitment	not applicable
Ethics oversight	not applicable

Note that full information on the approval of the study protocol must also be provided in the manuscript.

Field-specific reporting

Please select the one below that is the best fit for your research. If you are not sure, read the appropriate sections before making your selection.

[x] Life sciences [ ] Behavioural & social sciences [ ] Ecological, evolutionary & environmental sciences

For a reference copy of the document with all sections, see nature.com/documents/nr-reporting-summary-flat.pdf

Life sciences study design

All studies must disclose on these points even when the disclosure is negative.

Sample size	Sample size estimates have been performed on previous experience to obtain statistical significance and reproducibility. For in vitro experiments such as Western blot and flow cytometry, at least three samples were used per group for minimal statistics requirements. For in vivo studies, the sample size was determined to be sufficient to obtain the statistical difference between groups.
Data exclusions	No data were excluded from the analyses.
Replication	All experiments underlying main conclusions of this study have been successfully replicated multiple times and corroborated by several models. All the western blot and flow cytometry were carried out at least three times independently with the similar results.
Randomization	All samples/organisms were randomly allocated into experimental groups.
Blinding	No specific blinding was applied since all experiments were assigned into groups including relevalant controls and analysis was done objectively and without bias.

Reporting for specific materials, systems and methods

We require information from authors about some types of materials, experimental systems and methods used in many studies. Here, indicate whether each material, system or method listed is relevant to your study. If you are not sure if a list item applies to your research, read the appropriate section before selecting a response.

Materials & experimental systems

n/a	Involved in the study
	[x] Antibodies
	[x] Eukaryotic cell lines
[x]	Palaeontology and archaeology
	[x] Animals and other organisms
[x]	Clinical data
[x]	Dual use research of concern

Methods

n/a	Involved in the study
[x]	ChIP-seq
	[x] Flow cytometry
[x]	MRI-based neuroimaging

Antibodies

Antibodies used	Anti-CD86 (C-terminal) Polyclonal antibody (1: 1000, Proteintech, Rabbit mAb, #26903-1-AP)<br>Recombinant Anti-iNOS antibody (1: 1000, Abcam, Rabbit mAb, #ab178945)<br>Recombinant Anti-CD34 antibody (1: 200, Abcam, Rabbit mAb, #ab81289)<br>Recombinant Anti-Calreticulin antibody (1: 200, Abcam, Rabbit mAb, #ab92516)<br>Recombinant Anti-HMGB1 antibody (1: 200, Abcam, Rabbit mAb, #ab79823)<br>CD47 (D3O7P) Rabbit mAb (1:1000, Cell Signaling Technology, #63000)<br>Na,K-ATPase Antibody (1:1000, Cell Signaling Technology, #3010)<br>Integrin alpha 4/CD49D (1: 1000, Abcam, Rabbit mAb, #ab81280)<br>Integrin beta 1 (1: 1000, Abcam, Rabbit mAb, #ab52971)<br>Goat Anti-Rabbit IgG H&L (Alexa Fluor® 488) (1: 1000, Abcam, #ab150077)<br>Donkey Anti-Rabbit IgG H&L (Alexa Fluor® 647) (1: 1000, Abcam, #ab150075)<br>PE anti-mouse IFN-γ antibody (1: 200, Biolegend, #505807)<br>PE anti-mouse/human CD44 Antibody (1: 200, Biolegend, #103008)<br>PE/Cyanine7 anti-mouse CD62L Antibody (1: 200, Biolegend, #104418)<br>FITC anti-mouse CD11c Antibody (1: 200, Biolegend, #117306)<br>APC anti-mouse CD86 Antibody (1: 200, Biolegend, #105012)<br>PE anti-mouse CD80 Antibody (1: 200, Biolegend, #104708)<br>FITC anti-mouse CD3ε Antibody (1: 200, Biolegend, #100306)<br>Brilliant Violet 421™ anti-mouse CD4 Antibody (1: 200, Biolegend, #100438)<br>APC anti-mouse CD8a Antibody (1: 200, Biolegend, #100712)
Validation	For all the antibodies, we carried out western blot according to the method on the company's website, and detected whether the band size met the expectation with molecular weight marker, and added appropriate positive control and negative control. Antibodies purchased from Cell Signaling Technology and Abcam were validated as per their website stating "Antibody signal is measured in model systems with known presence/absence of target signal. Besides, each antibody's manual contains authentic data results from the companies (Biolegend, Abcam, Cell Signaling Technology, and Proteintech) validating specificity, and our data also verifies the corresponding antibody's specificity.

Eukaryotic cell lines

Policy information about cell lines and Sex and Gender in Research

Cell line source(s)	4T1, RAW 264.7, MCF-10A and HK-2 cells were purchased from Cell Bank of Shanghai, Chinese Academy of Sciences (Shanghai, China).
Authentication	The cell lines were certified by the manufacturers (surface markers, morphology).
Mycoplasma contamination	Cells were routinely screened for free of Mycoplasma contaminations. All cell lines are Mycoplasma negative with this study.
Commonly misidentified lines (See ICLAC register)	None of the cell lines used in this study is commonly misidentified cell line.

Animals and other research organisms

Policy information about studies involving animals; ARRIVE guidelines recommended for reporting animal research, and Sex and Gender in Research

Laboratory animals	Female BALB/c mice (7 weeks old) were purchased from Laboratory Animal Center of the Academy of Military Medical Sciences (Beijing, China). All procedures involving animal were conducted in accordance with the guidelines set by the Tianjin Committee of Use and Care of Laboratory Animals, and approved by the Animal Ethics Committee of Nankai University. All mice were cultured in suitable temperature and humidity environment (25 ℃, suitable humidity (typically 50 %), 12 hour dark/light cycle), and fed with sufficient water and food.
Wild animals	The study did not involve wild animals.

Reporting on sex | Because the selected model was breast cancer, female animals were selected for experiments. Although we have used single-sex animals in our research, we think that the research results were not only applicable to single sex.

Field-collected samples | The study did not involve samples collected from the filed.

Ethics oversight | All animal studies were conducted under the guidelines set by Tianjin Committee of Use and Care of Laboratory Animals, and the overall project protocols were approved by the Animal Ethics Committee of Nankai University.

Note that full information on the approval of the study protocol must also be provided in the manuscript.

Flow Cytometry

Plots

Confirm that:

- [x] The axis labels state the marker and fluorochrome used (e.g. CD4-FITC).
- [x] The axis scales are clearly visible. Include numbers along axes only for bottom left plot of group (a 'group' is an analysis of identical markers).
- [x] All plots are contour plots with outliers or pseudocolor plots.
- [x] A numerical value for number of cells or percentage (with statistics) is provided.

Methodology

Sample preparation	For flow cytometric analysis, on day 14, lymph nodes and tumors of 4T1-tumor-bearing mice received various treatments were collected and prepared to single cell suspensions by mechanical grinding. Subsequently, the cell suspensions were collected by centrifugation at 1,500 rpm for 5 min, and red blood cells (RBCs) were removed using modified RBC lysis buffer at room temperature for 5 min, followed by washing twice with 1×PBS. To investigate the proportions of mature DCs, the cell suspensions from lymph nodes of various groups were co-stained with anti-CD86-PE, anti-CD80-APC and anti-CD11c-FITC at room temperature in dark for 15 min, followed by flow cytometric analysis after washing three times with 1×PBS. The single cell suspensions collected from tumors with various treatments were co-incubated with anti-CD3-FITC and anti-CD8-APC for the analysis of the level of tumor-infiltrating CD8+ T cells at room temperature for 15 min, respectively, followed by flow cytometric analysis after washing three times with 1 × PBS. For studying the immune memory effect, Single cell suspensions from lymph nodes of 4T1-tumor-bearing mice were co-stained with anti-CD44-PE antibodies, anti-CD8-APC, anti-CD3-FITC, and anti-CD62L- PE/Cyanine7 for 15 min in dark at room temperature to investigate the effector memory T cells (Tem cells, CD3+CD8+CD44+CD62L-), and final analyses were conducted by flow cytometry after washing three times with 1×PBS.
Instrument	BD FACSAria III
Software	Data analysis: FlowJo version 10.0
Cell population abundance	No cell sorting was performed.
Gating strategy	Gating was first based on FSC/SSC and singlet cells were gated for further analysis. The cell populations were then analyzed based on expression of markers. Gating was then based on positive level.

- [x] Tick this box to confirm that a figure exemplifying the gating strategy is provided in the Supplementary Information.
